# USP-ddG: a unified structural paradigm with data efficacy and mixture-of-experts for predicting mutational effects on protein–protein interactions

**DOI:** 10.1093/bioinformatics/btag249

**Published:** 2026-07-07

**Authors:** Guanglei Yu, Xuehua Bi, Qichang Zhao, Jianxin Wang

**Affiliations:** School of Computer Science and Engineering, Central South University, Changsha 410083, China; Hunan Provincial Key Lab on Bioinformatics, Central South University, Changsha 410083, China; College of Medical Engineering and Technology, Xinjiang Medical University, Urumqi 830017, China; College of Medical Engineering and Technology, Xinjiang Medical University, Urumqi 830017, China; School of Computer Science and Engineering, Central South University, Changsha 410083, China; Hunan Provincial Key Lab on Bioinformatics, Central South University, Changsha 410083, China; School of Computer Science and Engineering, Central South University, Changsha 410083, China; Hunan Provincial Key Lab on Bioinformatics, Central South University, Changsha 410083, China

## Abstract

**Motivation:**

Accurately estimating changes in binding free energy (ΔΔG) is critical for understanding protein–protein interactions (PPIs) and guiding rational protein design. Recent deep learning methods have achieved notable progress by pre-training on large-scale structural data. While some approaches explore structural flexibility through energy-based sampling or generative modeling, these strategies typically involve substantial computational cost and overlook data efficacy in terms of training data organization.

**Results:**

We present USP-ddG, a unified structural paradigm for ΔΔG prediction built on a dual-channel architecture. The model incorporates three complementary components: (i) an inverse folding-based log-odds ratio, (ii) the empirical force field FoldX capturing side-chain packing energetics, and (iii) a geometric encoder that leverages Gaussian coordinate perturbation as a regularization strategy to improve robustness. To enhance representation capacity, we introduce a framework that integrates feed-forward network (FFN) and Mixture-of-Experts (MoE) to model domain-invariant and -specific features, respectively. We further propose CATH-guided Folding Ordering (CFO), a data efficacy strategy that organizes samples to mitigate catastrophic forgetting and data distribution bias. USP-ddG consistently outperforms existing state-of-the-art methods on the SKEMPI v2.0 benchmark, including the challenging hold-out CATH test set. It achieves superior accuracy on both single- and multi-point mutations and demonstrates strong performance in antibody affinity optimization against H1N1 and HER2, and in predicting the impact of SARS-CoV-2 variants on hACE2 binding. Ablation studies confirm the contribution of each component. These results highlight USP-ddG as a robust and data-efficient framework for modeling mutational effects on PPIs.

**Availability and implementation:**

USP-ddG is available at https://github.com/ak422/USP-ddG.

## 1 Introduction

As the ultimate executors of cellular structures and functions, proteins communicate through specific physical interactions and assemble into molecular machines, participating in nearly all cellular activities (Kastritis and Bonvin 2013, Greenblatt *et al.* 2024). Mechanistic dissection of PPIs has provided a foundation for engineering therapeutics that aim at treating, e.g. amyloid-related diseases (Eisenberg and Jucker 2012, Härd and Lendel 2012) and cancers (Lapenna and Giordano 2009, Schreiber *et al.* 2011).

The binding between protein molecules can be regarded as a reversible and rapid equilibrium process, with the binding strength typically quantified by thermodynamic parameters, such as equilibrium dissociation constant ($K_{D}$) or binding free energy (ΔG). Thus, developing methods to predict the changes in binding free energy (ΔΔG) caused by amino acid mutations is of critical importance in protein design (Mandell and Kortemme 2009). This involves screening mutant sequences to identify optimized variants that significantly reduce ΔG and thereby enhance binding strength. However, the potential sequence space of mutations is extremely large, and conventional wet-lab experiments face significant challenges due to limited throughput and high costs. In this context, the development of computational methods for accurate and efficient ΔΔG prediction has become an urgent need (Marchand *et al.* 2022).

Currently, a variety of computational approaches have been developed for predicting ΔΔG. However, traditional methods based on physical energy functions and empirical force fields, as well as experimental techniques, often suffer from inherent limitations such as operational complexity and low throughput. Among the state-of-the-art (SoTA) computational protocols, flex ddG in the Rosetta suite estimates ΔΔG through sampling conformational ensembles and incorporating the “backrub” algorithm to account for protein conformational plasticity (Barlow *et al.* 2018). Although physically rigorous, this method suffers from low computational efficiency, lagging behind deep learning approaches by orders of magnitude (Bushuiev *et al.* 2024, Yu *et al.* 2025). In contrast, FoldX utilizes its empirical effective energy function (EEEF) to rapidly and quantitatively assess the effects of mutations (ΔΔG) (Guerois *et al.* 2002, Schymkowitz *et al.* 2005, Delgado *et al.* 2019). The parameters of this energy function are optimized through fitting to large-scale experimental datasets using machine learning techniques, rendering it highly reliable for protein engineering applications. Recently, energy terms derived from FoldX have been integrated as effective prior knowledge into deep neural networks, enabling fast and accurate prediction of ΔΔG (Yu *et al.* 2025).

Recent advances have enabled the development of methods for predicting ΔΔG directly from sequences. MuLAN (Lombardi and Carbone 2024) leverages a protein language model (PLM) and light attention mechanisms to estimate ΔΔG without requiring structural information. While such methods offer scalability and applicability to proteins lacking resolved structures, they are limited in capturing structural context and conformational flexibility. In contrast, structure-based pre-trained models have been developed to learn generalizable representations from 3D structures. These approaches typically reconstruct masked residues or local properties from structural environments, capturing geometric and physicochemical patterns. Notable examples include (Anand *et al.* 2022, Mohseni Behbahani *et al.* 2023, Pun *et al.* 2024).

With the rapid advancement of deep learning, data-driven approaches have emerged as promising alternatives for ΔΔG prediction (Shan *et al.* 2022, Wang *et al.* 2023, Yu *et al.* 2025). Despite continuous improvements, predictive performance remains constrained by the scarcity of high-quality experimental annotations. To address this limitation, self-supervised pre-training on large-scale unlabeled structural data has become a prevalent paradigm for enhancing model generalization. Common proxy tasks for pre-training include diverse learning objectives, such as inverse folding modeling (Yang *et al.* 2020), atomic coordinate reconstruction (Liu *et al.* 2021), side-chain conformation prediction (Liu *et al.* 2023, Luo *et al.* 2023), and masked language modeling (Wu *et al.* 2024). These pre-training strategies enable the learning of sequence and structure representations imbued with physicochemical constraints, while highlighting the challenge of transferring such prior knowledge to downstream ΔΔG prediction (Jiao *et al.* 2025).

In response, and under the assumption of fixed backbone upon mutations, BA-DDG estimates ΔΔG using the Boltzmann distribution, incorporating the difference in sequence negative log-likelihood scores derived from inverse folding model ProteinMPNN (Dauparas *et al.* 2022), thereby achieving substantially improved predictive accuracy (Jiao *et al.* 2025). In contrast, EBM-DDG relaxes the fixed backbone assumption to accommodate structural rearrangements upon mutations. It decomposes ΔΔG into a sequence term, derived from BA-DDG, and a structural term. The structural term is estimated using the diffusion-based score-matching model DSMBind (Jin *et al.* 2023), which generates mutant conformations and quantifies associated energy changes, enabling more comprehensive ΔΔG modeling (Soga *et al.* 2025). However, it overlooks the conformational dynamics of proteins, and focuses on backbone-only determinants of ΔΔG, which may result in the loss of critical information contributed by conformational dynamics and side-chains.

In this work, we propose USP-ddG, a unified structural paradigm for predicting ΔΔG on PPIs. The model integrates both sequence and structure as input and decomposes ΔΔG into three components with an auxiliary task. The overall architecture of USP-ddG is as follows:

1. Component 1: leveraging the thermodynamic cycle definition, we use the inverse folding model ProteinMPNN to compute sequence negative log-likelihood scores, enabling USP-ddG to learn the correlations between residue preferences and mutational effects.
2. Component 2: this component focuses on ΔΔG prediction with energy terms from FoldX, enabling the model to learn physical inductive bias from empirical data to account for thermodynamic properties of proteins.
3. Component 3: we use an improved CATH-ddG of our previous work for encoding the protein sequence and 3D structure. To improve model robustness against minor structural perturbations, Gaussian noise is added to atomic coordinates during training as a form of data augmentation and regularization. Specifically, we introduce a hybrid architecture that integrates the standard fully connected Feed-Forward Network (FFN) layer (Yu *et al.* 2025) and MoE to capture domain-invariant and -specific features, respectively. In addition, we propose CATH-guided Folding Ordering (CFO), a curriculum learning-based strategy to enhance learning efficiency by optimizing the ordering of training data. Unlike random shuffling, CFO aims at mitigating issues such as catastrophic forgetting and data distribution bias.
4. Integration of CATH domain alignment: to integrate protein domain classification information for better representation learning, we formulate the structural constraints derived from CATH domains as an auxiliary multi-label classification task within a self-supervised learning (SSL) framework, enabling the model to learn the structural clusters and functional families within its CATH superfamilies.

We conduct extensive experiments to validate the effectiveness of USP-ddG. Specifically, we adopt the hold-out CATH test set introduced in CATH-ddG (Yu *et al.* 2025), which is curated from SKEMPI v2.0 (Jankauskaite *et al.* 2019). This independent test set contains 813 automatically constructed mutations, which ensures no overlap in CATH superfamily with the training set while also maintaining a maximum TM-score <0.6 for structural similarity. Our results demonstrate that USP-ddG consistently achieves the best performance. In addition, ablation studies confirm the critical contributions of data efficacy and MoE strategies, as well as the use of inverse folding and improved CATH-ddG in enhancing model performance. Overall, the results strongly support the robustness and superior effectiveness of USP-ddG in predicting ΔΔG. Independent case studies demonstrate successful enhancement of binding affinity on antibody variants of CR6261 against H1N1 influenza A, trastuzumab against human epidermal growth factor receptor 2 (HER2), and variants in receptor-binding domain (RBD) of SARS-CoV-2 to human angiotensin-converting enzyme 2 (hACE2) receptor.

## 2 Materials and methods

### 2.1 Notation and problem definition

A protein chain A is represented by *n* amino acids $S_{A}\in {\left\{1,\ldots ,20\right\}}^{n}$, and its atomic coordinates $X_{A}\in R^{n\times m\times 3}$. Here, *m* represents the number of atoms per amino acid. A protein complex AB is formed by the binding of two chains, A and B, and exists in a dynamic equilibrium between its bound state $AB_{\text{bnd}}$ and unbound state $AB_{\text{unbnd}}$. We refer to the unmutated protein chain as the wild-type (wt) and the mutated chain as the mutant (mut). For simplicity, this section focuses on two-chain complexes, using the notation $\left\{S_{AB},X\right\}$ to represent the sequence and its corresponding structure.

The binding free energy (ΔG) of a protein complex is defined as the difference in Gibbs free energy between the bound state ($G_{\text{bnd}}$) and the unbound state ($G_{\text{unbnd}}$): $\Delta G=G_{\text{bnd}}-G_{\text{unbnd}}$. Based on this definition, the change in binding free energy (ΔΔG) upon amino acid mutation is given by:


(1) $$\Delta \Delta G=\Delta G^{\text{mut}}-\Delta G^{\text{wt}},$$

where $\Delta G^{\text{mut}}$ and $\Delta G^{\text{wt}}$ represent the binding free energies of the mutant $\left\{S_{AB}^{\text{mut}},X^{\text{mut}}\right\}$ and wild-type $\left\{S_{AB}^{\text{wt}},X^{\text{wt}}\right\}$ complexes, respectively. Our objective is to predict ΔΔG for a given set of mutations {wt→mut}.

This study is grounded in prior work that reveals the intrinsic relationship between ΔΔG and protein inverse folding. The details on how to estimate ΔΔG using Boltzmann distribution with the negative log-likelihood ratio can be found in Section A, available as supplementary data at *Bioinformatics* online.

### 2.2 Overview

**Figure 1:**
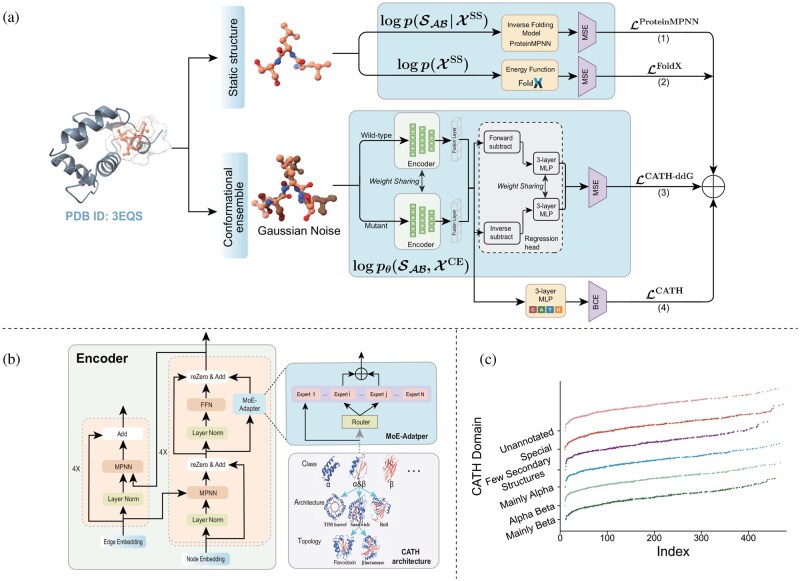
(a) Overview of the proposed USP-ddG framework. The framework is composed of a dual-channel and four task branches: (1) inverse folding-based supervised learning task, (2) energy function-based supervised learning task, (3) self-supervised learning task for CATH domains, and (4) training with structure noise-based supervised learning task. (b) To utilize the advantage of sparsely activated of MoE to capture the shared and distinctive features across diverse protein domains, we improved the Feed-Forward Network via FFN with MoE to learn domain-invariant and specific features of protein domains. (c) Illustration of CATH-guided Folding Ordering. These results are based on simulated data.

Figure 1a illustrates the overall architecture of USP-ddG. The framework comprises two complementary channels. (i) The static-structure (SS) channel decomposes ΔΔG into an inverse folding term derived from ProteinMPNN and a structural energy term computed using FoldX. (ii) Conformational-ensemble (CE) channel extends our previous work CATH-ddG by incorporating an auxiliary SSL task that aligns latent embeddings with protein domain-induced preferences, thereby improving both predictive performance and interpretability in downstream ΔΔG prediction. Finally, the overall loss is defined as a learnable weighted sum of the supervised prediction and SSL tasks.

### 2.3 Modeling with conformational-ensemble assumptions

Inverse folding-based models for predicting ΔΔG (e.g. BA-DDG and EBM-DDG) typically rely on two assumptions: (i) protein function is primarily dictated by backbone atoms, and (ii) the protein exists in a unique, static structure. However, protein function is orchestrated by a complex dynamic conformational landscape involving coupled motions and flexibility of both the backbone and side-chains. Consequently, prior approaches relying exclusively on the backbone fail to capture the full physicochemical complexity. Thus, we propose a unified ΔΔG prediction framework that incorporates side-chains and dynamic conformations into the structural assumptions. To formalize this as a tractable computational approximation, we correspondingly define ΔΔG as:


(2) $$\Delta \Delta G=\Delta \Delta G^{\text{SS}}+\Delta \Delta G^{\text{CE}},$$

where $\Delta \Delta G^{\text{SS}}$ and $\Delta \Delta G^{\text{CE}}$ are defined under the static-structure and conformational-ensemble hypotheses, respectively.

**Static-structure assumption (** $\Delta G^{\text{SS}}$ **)**. Under the static-structure hypothesis, the binding free energy $\Delta G^{\text{SS}}$ can be derived from the Boltzmann distribution (Atkins *et al.* 2023) and is expressed in terms of the probabilities of the bound and unbound state, denoted $p_{\text{bnd}}^{\text{SS}}$ and $p_{\text{unbnd}}^{\text{SS}}$, respectively, as shown below:


(3) $$\begin{array}{cc} & \Delta G^{\text{SS}}=G_{\text{bnd}}^{\text{SS}}-G_{\text{unbnd}}^{\text{SS}}=-k_{B}T\cdot \left(\text{log}\;p_{\text{bnd}}^{\text{SS}}-\text{log}\;p_{\text{unbnd}}^{\text{SS}}\right)\\ & \;=-k_{B}T\cdot \left(\text{log}\;p(X_{\text{bnd}}^{\text{SS}},S_{AB})-\text{log}\;p(X_{\text{unbnd}}^{\text{SS}},S_{AB})\right). \end{array}$$

Following the detailed derivation presented in Section B, available as supplementary data at *Bioinformatics* online, we obtain the binding free energy $\Delta G^{\text{SS}}$ under the static-structure assumption, which is formulated as:


(4) $$\Delta G^{\text{SS}}=\Delta G_{\text{ProteinMPNN}}+\Delta G_{\text{FoldX}}.$$

**Conformational-ensemble assumption (** $\Delta G^{\text{CE}}$ **)**. Under the conformational-ensemble hypothesis, the binding free energy is formally defined as the difference between the bound and unbound conformational ensembles. Thermodynamically rigorous sampling of conformational ensembles is computationally prohibitive. In this work, we adopt a tractable modeling approximation for mutational effects using structural perturbations implemented as Gaussian coordinate noise around the bound state, under the assumption that unbound state contributions largely cancel out in ΔΔG calculations, leading to the following practical formulation:


(5) $$\begin{array}{cc} \Delta G^{\text{CE}} & =G_{\text{bnd}}^{\text{CE}}-G_{\text{unbnd}}^{\text{CE}}=-k_{B}T\cdot \;\text{log}\;p_{\theta }(X_{\text{bnd}}^{\text{CE}},S_{AB})\\ & ≜\Delta G_{\text{CATH-ddG}}. \end{array}$$

where θ is parameterized by an improved version of our previous model, CATH-ddG, denoted as $\Delta G_{\text{CATH-ddG}}$. The model takes the sequence $S_{AB}$ and the structure $X_{\text{bnd}}^{\text{CE}}$ as input, with Gaussian coordinate perturbation applied as a regularization strategy to improve robustness. The detailed architecture of the improved CATH-ddG and the procedure for applying Gaussian noise are presented in Sections C and D, available as supplementary data at *Bioinformatics* online, respectively.

### 2.4 Unified structural paradigm

Under the preceding assumptions, we use a unified structural paradigm, named USP-ddG, to evaluate ΔΔG by integrating the derived components. Note that additive decomposition of ΔG is adopted as a modeling approximation to combine complementary information from different predictive components. As shown in Table 4, removing individual components leads to consistent degradation in predictive performance, providing empirical support for this formulation. Accordingly, the overall binding free energy ΔG can be expressed as follows:


(6) $$\begin{array}{cc} \Delta G & =\Delta G^{\text{SS}}+\Delta G^{\text{CE}}\\ & =\Delta G_{\text{ProteinMPNN}}+\Delta G_{\text{FoldX}}+\Delta G_{\text{CATH-ddG}}, \end{array}$$

where the first term is predicted by the pre-trained inverse folding model ProteinMPNN, the second term is calculated using FoldX, and the third term is predicted by the improved, trainable neural network model CATH-ddG. Finally, the overall ΔΔG is then expressed as:


(7) $$\begin{array}{cc} \Delta \Delta G & =\left(\Delta G_{\text{ProteinMPNN}}^{\text{mut}}+\Delta G_{\text{FoldX}}^{\text{mut}}+\Delta G_{\text{CATH-ddG}}^{\text{mut}}\right)\\ & \;-\left(\Delta G_{\text{ProteinMPNN}}^{\text{wt}}+\Delta G_{\text{FoldX}}^{\text{wt}}+\Delta G_{\text{CATH-ddG}}^{\text{wt}}\right). \end{array}$$

Following Yu *et al.* (2025), we incorporate an auxiliary self-supervised loss to align with the inductive biases of protein domains. The final objective function combines the Mean Squared Error (MSE) loss and Binary Cross-Entropy (BCE) loss as follows:


(8) $$\begin{array}{c} L=L^{\text{ProteinMPNN}}+L^{\text{FoldX}}+L^{\text{CATH-ddG}}+L^{\text{CATH}}\\ \;=\lambda_{1}\text{MSE}(\Delta \Delta G_{\text{ProteinMPNN}},\Delta \Delta G)\\ \;+\lambda_{2}\text{MSE}(\Delta \Delta G_{\text{FoldX}},\Delta \Delta G)\\ \;+\lambda_{3}\text{MSE}(\Delta \Delta G_{\text{CATH-ddG}},\Delta \Delta G)\\ \;+\lambda_{4}\text{BCE}({\hat{y}}_{\text{CATH}},y_{\text{CATH}}), \end{array}$$

where ${\left\{\lambda_{i}\right\}}_{i=1}^{4}$ are learnable scalar parameters, as shown in Fig. 1a.

**Table 4:** Impact of individual module exclusion on performance drop in the hold-out CATH test set. The bold values denote the best results for each metric.

Model	Mutations	Overall					Per-PPI	
		**PearsonR** ↑	**SpearmanR** ↑	**RMSE** ↓	**MAE** ↓	**AUROC** ↑	**PearsonR** ↑	**SpearmanR** ↑
w/o MoE	All	0.639	0.625	2.000	1.471	0.786	0.447	0.412
	Single	0.580	0.559	1.592	1.197	0.757	0.509	0.438
	Multiple	0.652	0.591	2.606	2.022	0.824	0.653	0.647
w/o inverse folding	All	0.627	0.606	2.027	1.506	0.778	0.496	0.460
	Single	0.558	0.529	1.623	1.217	0.731	0.533	0.480
	Multiple	0.646	0.555	2.625	2.080	0.853	0.606	0.576
w/o CATH-ddG	All	0.596	0.618	2.087	1.529	0.784	0.471	0.396
	Single	0.552	0.553	1.630	1.223	0.749	0.512	0.433
	Multiple	0.589	0.550	2.778	2.162	0.831	0.680	0.655
w/o sampler	All	0.655	0.635	1.965	1.456	0.794	**0.542**	**0.505**
	Single	0.602	0.578	1.561	1.168	0.761	0.584	0.519
	Multiple	0.664	0.577	2.570	2.029	0.838	0.671	0.653
USP-ddG	All	**0.677** ± 0.022	**0.654** ± 0.024	**1.914** ± 0.057	**1.422** ± 0.044	**0.802** ± 0.020	**0.542** ± 0.067	0.493 ± 0.063
	Single	**0.629** ± 0.032	**0.596** ± 0.033	**1.520** ± 0.056	**1.140** ± 0.044	**0.772** ± 0.025	**0.602** ± 0.035	**0.536** ± 0.051
	Multiple	**0.689** ± 0.037	**0.611** ± 0.043	**2.492** ± 0.098	**1.990** ± 0.092	**0.848** ± 0.035	**0.695** ± 0.072	**0.668** ± 0.088

### 2.5 Training with mixture-of-experts

We leverage sparse MoE (Shazeer *et al.* 2017) to establish a scalable framework for mitigating the issue of structural overlaps across different CATH superfamilies (Nallapareddy *et al.* 2023). This section first outlines the MoE mechanism and then details its integration into the FFN layer.

The MoE contains two components: the *N* experts ${\left\{E_{i}\right\}}_{i=1}^{N}$ and a routing network R. While it maintains efficiency by activating only a subset of experts for each input token, its output is combined dynamically via a weighted strategy by the router R. Inspired by Yu *et al.* (2024), we use the LoRA (Hu *et al.* 2022) adapter as the expert in MoE to speed up the adaptation on downstream tasks. Our MoE-Adapters are implemented in all structured Transformer blocks of the spatial and sequential encoders of CATH-ddG. Formally, let $x\in R^{L_{\text{patch}}\times d_{\text{model}}}$ be the input tokens. The combined output $h_{\mathrm{MoE}}\in R^{L_{\text{patch}}\times d_{\text{model}}}$ is then computed as:


(9) $$h_{\mathrm{MoE}}=\sum_{i=1}^{N}\omega_{i}E_{i}(x),$$

where $w=[\omega_{1},\omega_{2},\ldots ,\omega_{N}]\in R^{L_{\text{patch}}\times N}$ represents the gating weights assigned by router R, dictating each expert’s contribution to the output $h_{\mathrm{MoE}}$. The gating weights are computed using a learnable matrix $W\in R^{N\times d_{\text{model}}}$ and a learnable matrix for the noise component $W_{\text{noise}}\in R^{N\times d_{\text{model}}}$ as follows (Shazeer *et al.* 2017):


(10) w=Softmax(TopK(H(x))),

(11) $$H(x)=\mathrm{Wx}+\text{StandardNormal}()\cdot \text{Softplus}\left(W_{\text{noise}}x\right).$$

For FFN layer, we adopt an architecture inspired by DeepSeek-MoE (Liu *et al.* 2024), which utilizes a hybrid layer of shared and routed experts. Specifically, the shared expert is dedicated to learning domain-invariant knowledge and reducing redundancy among the routed experts, while the routed experts specialize in domain-specific knowledge of protein structures. Thus, the output of FFN layer after the attention head, denoted as $h_{\mathrm{FFN}}^{\prime }\in R^{L_{\text{patch}}\times d_{\text{model}}}$, is redefined as:


(12) $$h_{\mathrm{FFN}}^{\prime }=x+m⊙(\alpha \cdot \text{Linear}\left(\text{Concat}\left(h_{\mathrm{FFN}},h_{\mathrm{MoE}}\right)\right)),$$

where m∼Bernoulli(p=0.1) is the dropout mask vector, ⊙ is the Hadamard product operation, α is a learnable scalar for reZero (Bachlechner *et al.* 2021), and Concat(·) represents the concatenation operation, as shown in Fig. 1b.

### 2.6 Training with data efficacy

Machine learning models typically achieve reliable performance when the training data accurately capture the real-world distributions. However, even SoTA algorithms often suffer from limited generalization performance when deployed in distribution shifts (Ta and Stokes 2025, Ursu *et al.* 2025). This raises a critical question: how can we systematically enhance model performance without altering the data scale or model architecture? The answer may lie not in the data itself, but rather in how it is utilized. Currently, data efficiency aims at curating data in terms of both quantity and quality to improve model performance. In contrast, data efficacy is concerned with maximizing performance by optimizing the organization and utilization of training data (Dai *et al.* 2025).

Additionally, investigators have demonstrated that while easy negative samples promote shortcut learning, hard negatives are essential for compelling models to learn complex features, thereby ultimately facilitating the discovery of biological mechanistic principles (Robinson *et al.* 2021, Ursu *et al.* 2025). To build models that generalize across diverse real-world scenarios, it is crucial to challenge them with appropriately difficult data. To this end, we propose the CATH-guided Folding Ordering (CFO) strategy, which organizes training data by leveraging evolutionary insights from CATH protein domain annotations to present increasingly difficult samples. More specifically, to construct the training set enriched with hard negatives, we partition the training data into Class-disjoint subsets according to the Class-level categorical definition in the CATH database. The permutation function $\pi_{\text{CFO}}$, which defines the training order, is formalized as follows:


(13) $$\pi_{\text{CFO}}(D;L)=\overset{L-1}{\underset{\ell =0}{\cup }}<\pi_{\ell }|f(\pi_{\ell })=\mathrm{CATH}[\ell ],\pi_{\ell }\subseteq D>\;\;$$

where $f(\pi_{\ell })$ returns the Class of $\pi_{\ell }$, CATH denotes the set of Class-level categories containing an unannotated entry (Yu *et al.* 2025), and L=|CATH|. Thus, based on the Class distributions, an illustration of CFO is provided in Fig. 1c, which starts with easier samples and progressively tackles harder ones.

## 3 Experimental settings

This section introduces the experimental settings, including the datasets and baseline methods used for comparison. Comprehensive details regarding the evaluation metrics and parameter settings are provided in Section E and F, available as supplementary data at *Bioinformatics* online, respectively.

### 3.1 Datasets

To train and evaluate USP-ddG, we utilize the largest available curated dataset, SKEMPI v2.0, which comprises 7085 mutations. Previous studies have primarily benchmarked models using a PPI-disjoint cross-validation split on this dataset (Liu *et al.* 2023, Luo *et al.* 2023, Jiao *et al.* 2025, Soga *et al.* 2025). However, this evaluation strategy is susceptible to data leakage and may overestimate model performance. As demonstrated in our prior work (Yu *et al.* 2025), an average of 88.70% of mutation entries under this split are classified as easy mutations—defined as those exhibiting a maximum TM-score≥0.6 when aligned to structures in the training set. To enable a more rigorous and functionally relevant assessment, we adopt a partitioning strategy based on CATH homologous superfamilies defined in CATH-ddG (Yu *et al.* 2025). Thus, SKEMPI v2.0 is divided such that the hold-out CATH test set contains only complexes that share no CATH superfamilies with the training set. This procedure yields a challenging test set of 813 mutations, in which none are classified as easy mutations, offering a robust evaluation of model generalization toward unseen CATH homologous superfamilies (see Section G, available as supplementary data at *Bioinformatics* online for an overview of data splitting).

### 3.2 Baselines

For the hold-out CATH test set selected from SKEMPI v2.0, we evaluated two versions of our method, ${\text{USP-ddG}}^{\text{Random}}$ (trained on randomly shuffled data) and USP-ddG (trained with $\pi_{\text{CFO}}$ guidance), against three categories of SoTA baselines, utilizing retrained and retested results whenever available. To ensure a comprehensive and fair evaluation, we select baselines representing diverse methodological paradigms with publicly available tools or source code: (i) traditional energy functions [flex ddG (Barlow *et al.* 2018) and FoldX (Delgado *et al.* 2019)]; (ii) pre-training based approaches [RDE-Network (Luo *et al.* 2023) and DiffAffinity (Liu *et al.* 2023)]; and (iii) supervised learning approaches [CATH-ddG (Yu *et al.* 2025) and BA-DDG (Jiao *et al.* 2025)].

## 4 Results

We evaluate our method using both random shuffling (denoted as ${\text{USP-ddG}}^{\text{Random}}$) and the proposed CFO-based data efficacy strategy (denoted as USP-ddG), benchmarking it against previous works. Performance of USP-ddG is reported as the mean ± standard deviation across ensemble models. Statistical significance of improvements over CATH-ddG is assessed using two-sided bootstrap resampling (*B* = 5000), with significant results (*P* < .05) marked by asterisks (*) in Tables 1–4 and Table 1, available as supplementary data at *Bioinformatics* online. In the following performance evaluation, **bold** and underlined values denote the best and second-best results for each metric, respectively. Additional results for USP-ddG on the binding of SARS-CoV-2 RBD to hACE2 are provided in Section H, available as supplementary data at *Bioinformatics* online.

**Table 1:** Performance on the hold-out CATH test set under all-, single-, multi-point mutations. The bold and underlined values denote the best and second-best results for each metric.

Category	Method	Mutations	Overall					Per-PPI	
			**PearsonR** ↑	**SpearmanR** ↑	**RMSE** ↓	**MAE** ↓	**AUROC** ↑	**PearsonR** ↑	**SpearmanR** ↑
Energy function	FoldX	All	0.460	0.525	2.308	1.684	0.754	0.493	0.430
		Single	0.451	0.492	1.745	1.302	0.713	0.500	0.446
		Multiple	0.403	0.370	3.146	2.429	0.835	0.583	0.548
	flex ddG	All	0.608	0.610	2.064	1.495	0.764	0.508	0.454
		Single	0.570	0.533	1.607	1.200	0.707	0.568	0.463
		Multiple	0.620	0.562	2.697	2.054	0.828	0.616	0.640
Pre-training based	RDE-network	All	0.459	0.470	2.310	1.703	0.745	0.350	0.288
		Single	0.383	0.376	1.806	1.355	0.690	0.347	0.253
		Multiple	0.450	0.379	3.070	2.403	0.820	0.503	0.473
	DiffAffinity	All	0.314	0.363	2.468	1.739	0.625	0.323	0.249
		Single	0.264	0.293	1.886	1.384	0.594	0.336	0.250
		Multiple	0.246	0.260	3.332	2.443	0.673	0.290	0.288
Supervised learning	CATH-ddG	All	0.615	0.627	2.050	1.505	0.781	0.526	0.494
		Single	0.625	0.594	1.526	**1.138**	0.746	0.569	0.509
		Multiple	0.575	0.527	2.813	2.252	0.833	0.596	0.551
	BA-DDG	All	0.414	0.496	2.365	1.696	0.732	0.464	0.402
		Single	0.446	0.482	1.750	1.275	0.711	0.502	0.423
		Multiple	0.336	0.309	3.234	2.442	0.739	0.660	0.618
	${\text{USP-ddG}}^{\text{Random}}$	All	0.655	0.635	1.965	1.456	0.794	**0.542**	**0.505**
		Single	0.602	0.578	1.561	1.168	0.761	0.584	0.520
		Multiple	0.664	0.577	2.570	2.029	0.838	0.671	0.653
	USP-ddG	All	**0.677** ± 0.022^a^	**0.654** ± 0.024^a^	**1.914** ± 0.057^a^	**1.422** ± 0.044^a^	**0.802** ± 0.020^a^	**0.542** ± 0.067	0.493 ± 0.063
		Single	**0.629** ± 0.032	**0.596** ± 0.033	**1.520** ± 0.056	1.140 ± 0.044	**0.772** ± 0.025	**0.602** ± 0.035	**0.536** ± 0.051
		Multiple	**0.689** ± 0.037^a^	**0.611** ± 0.043^a^	**2.492** ± 0.098^a^	**1.990** ± 0.092^a^	**0.848** ± 0.035	**0.695** ± 0.072	**0.668** ± 0.088

a *P* values are for comparison with CATH-ddG; *P* < .05.

**Table 2:** Evaluation of the binding affinity of CR6261 against H1N1. The bold values denote the best results for each metric.

Category	Method	**PearsonR** ↑	**SpearmanR** ↑
Energy function	FoldX	0.577	0.593
	flex ddG	0.515	0.516
Pre-training based	RDE-Network	0.370	0.422
	DiffAffinity	0.351	0.323
Supervised learning	CATH-ddG	0.499	0.502
	BA-DDG	0.583	0.566
	${\text{USP-ddG}}^{\text{Random}}$	0.608	0.584
	USP-ddG	**0.658** ±0.010 ^a^	**0.627** ±0.011 ^a^

a *P* values are for comparison with CATH-ddG; *P* < .05.

**Table 3:** Evaluation on the binding affinity of HER2 binders. The bold values denote the best results for each metric.

Category	Method	**PearsonR** ↑	**SpearmanR** ↑
Energy function	FoldX	0.246	0.329
	flex ddG	0.301	0.339
Pre-training based	RDE-Network	0.465	0.481
	DiffAffinity	0.590	0.524
	GearBind+Ensemble^a^	0.579	0.608
Supervised learning	CATH-ddG	0.607	0.639
	BA-DDG	0.326	0.362
	${\text{USP-ddG}}^{\text{Random}}$	0.619	0.656
	USP-ddG	**0.630** ± 0.027	**0.661** ± 0.027

a Results are from GearBind (Cai *et al.* 2024).

### 4.1 Performance comparison on SKEMPI v2.0 dataset

We evaluate our USP-ddG deep learning framework on the hold-out CATH test set curated from SKEMPI v2.0, and present the results in Table 1 for three categories of methods across seven evaluation metrics. The key findings are summarized below: (i) USP-ddG consistently outperforms all baselines on both single- and multi-point mutation subsets, achieving superior performance in 6 out of 7 metrics when evaluated across all mutations. (ii) On this non-superfamily leakage test set, traditional force field simulators (e.g. the SoTA flex ddG) are surpassed by USP-ddG not only in computational efficiency but also in predictive accuracy, see Section I and J, available as supplementary data at *Bioinformatics* online for efficiency analysis. (iii) Without utilizing any additional pre-training data, USP-ddG exceeds all pre-training based methods across all metrics, suggesting that our unified structural framework is intrinsically more effective for ΔΔG prediction than approaches leveraging external pre-training knowledge. (iv) USP-ddG achieves clear improvements over BA-DDG, the strongest baseline, which has substantially outperformed other methods under PPI-disjoint cross-validation. (v) Finally, USP-ddG demonstrates the largest gains on multi-point mutations, a challenging due to the complexity of epistatic effects, thereby highlighting its strong practical applicability.

### 4.2 Evaluation on the antibody CR6261 against H1N1

CR6261 is a broadly neutralizing antibody (bnAb) against influenza A. Phillips *et al.* (Phillips *et al.* 2021) systematically measured the $K_{D}$ of combinatorially complete mutational libraries for 14 heavy-chain mutations, reconstructing all possible evolutionary intermediates to the germline sequence. Leveraging these experimental data, AbBiBench curated a benchmark of 1887 mutants targeting A/New Caledonia/20/1999 (H1N1) for evaluating model performance in designing high-affinity antibodies (PDB ID: 3GBN) (Zhao *et al.* 2025). Crucially, none of these mutants appears in any public training databases, ensuring the absence of mutation-level data leakage and enabling reliable assessment of generalization capability. Accurately predicting ΔΔG for the variants of antibody CR6261 presents a challenge due to the prevalence of multi-point mutations and epistatic effects. Epistasis introduces non-additive interactions between mutations, which not only constrains the evolutionary paths accessible under selective pressure but also complicates the difficulty of accurately predicting ΔΔG. The results in Table 2 show that USP-ddG achieves the best performance measured by PearsonR and SpearmanR, with PearsonR and SpearmanR values exceeding the SoTA baseline BA-DDG by 12.86% and 10.78%, respectively, demonstrating its superior generalization ability in capturing epistatic effects.

### 4.3 Evaluation on the antibody trastuzumab against HER2

The HER2 binders test set, sourced from Cai *et al.* (2024) and Shanehsazzadeh *et al.* (2023) (PDB ID: 1N8Z), comprises 419 variants featuring *de novo* designed complementarity determining region (CDR) loops. These variants were computationally designed to enhance the quality and controllability of the trastuzumab antibody using generative AI and have been experimentally validated by surface plasmon resonance (SPR), thus establishing the reliability for benchmarking AI-based antibody design methods. The antibody trastuzumab variants exhibit a high average edit distance of 7.6, presenting a significant challenge for ΔΔG predictors which are primarily trained on the low edit distance SKEMPI v2.0 dataset (Cai *et al.* 2024). As shown in Table 3, USP-ddG achieves the best performance on this independent test set, with PearsonR and SpearmanR values that are 3.79% and 3.44% higher, respectively, than the SoTA baseline CATH-ddG. In contrast, traditional energy function methods such as flex ddG and FoldX are substantially outperformed by machine learning-based approaches on this HER2 binder test set.

### 4.4 Ablation study

To elucidate the contribution of each component in USP-ddG, we performed an ablation study on the hold-out CATH test set. The model was systematically evaluated by progressively deactivating individual modules, followed by retraining and assessment. As illustrated in Table 4, we observed consistent declines in correlation metrics, underscoring the importance of each module in achieving optimal performance. Our results indicate that the inverse folding $\text{log}\;p(S_{AB}|X^{\text{SS}})$ and CATH-ddG $\text{log}\;p_{\theta }(S_{AB},X^{\text{CE}})$ modules contribute nearly equally to model performance. Specifically, CATH-ddG tends to offer a slightly greater contribution in scenarios involving multiple-point mutations, whereas inverse folding contributes more in single-point mutation cases. In contrast, the impact of data efficacy module (w/o Sampler) is less pronounced than that of the MoE module. Nevertheless, we observed that incorporating data efficacy improves generalization, evidenced in the case studies discussed above, thereby confirming its positive effect on model robustness.

### 4.5 Domain specialization

**Figure 2:**
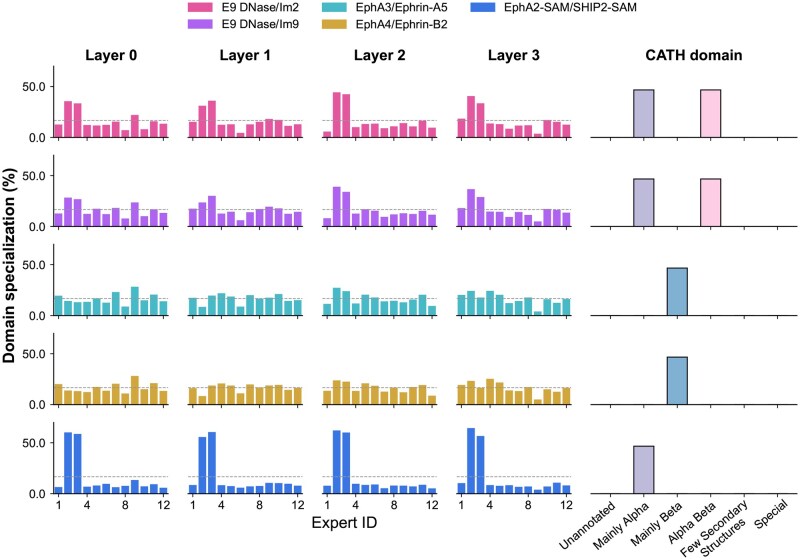
Domain specialization of USP-ddG. We visualize the routing distribution of tokens from different protein domains across each layer to the 12 experts for the five PPIs in the hold-CATH test set, considering the top-2 active experts per token. The horizontal gray dashed lines represent the uniform routing baseline of 16.67%  (2/12) per expert, which results from activating 2 out of 12 experts each token in every layer, providing reference for assessing the learned domain specialization.

Following Muennighoff *et al.* (2025), domain specialization characterizes the functional preference of expert $E_{i}$ for CATH domain $C_{j}$, representing the percentage of tokens from $C_{j}$ assigned to $E_{i}$, with 100% indicating exclusive dedication and 0% identifying prunable experts for that domain. Figure 2 reveals that many experts are significantly activated deviating from the uniform routing baseline on specific CATH domains. A notable example is the EphA2-SAM/SHIP2-SAM complex, where C-terminal sterile alpha motifs (SAM) form a heterodimer that negatively regulates receptor endocytosis and degradation (Vincenzi *et al.* 2024), resulting in the 2nd and 3rd experts in each layer specialize over 50%. This reflects that the domain specialization in USP-ddG results in low functional overlap, with dedicated experts exhibiting distinct domain preferences, thereby effectively alleviating structural overlaps among protein domains as intended. In addition, as the E9 DNase/Im9 and E9 DNase/Im2 complexes share similar CATH domains mediated by structurally analogous PPIs, they exhibit closely aligned domain specializations. Likewise, EphA3/ephrinA5 and EphA4/ephrinB2 PPIs also exhibit similar domain specializations. Consequently, as shown in Fig. 2 below, the MoE framework demonstrates the ability to precisely delineate these domain-specific features, this highlights its advantage in identifying determinants of PPIs and showcasing its potential to advance the development of protein domain representation learning.

## 5 Conclusion

Predicting ΔΔG for PPIs is a critical computational task in understanding proteins as the primary executors of cellular functions. Current methods often lack a systematic treatment of mutation-induced conformational changes and rarely adopt a unified structural perspective. In this work, we present USP-ddG, a unified structural framework that incorporates complementary static-structure and conformational-ensemble perspectives, offering an alternative view for binding free energy modeling. By integrating these perspectives via a hybrid layer of FFN and MoE, coupled with a data efficacy training strategy, USP-ddG outperforms existing approaches and provides a robust tool for ΔΔG prediction.

A qualitative comparison of ΔΔG prediction methods reveals that existing approaches can be broadly categorized by physical interpretability, computational cost, and data dependency. Energy function methods offer interpretability and generalizability but incur substantial computational costs. Pre-training based methods learn structural representations from large-scale protein data yet typically require task-specific fine-tuning. As a supervised learning method, USP-ddG directly optimizes predictive performance by integrating sequence and structural information with Gaussian perturbation regularization, achieving SoTA performance while maintaining computational efficiency and interpretability grounded in protein structural domain information. For a discussion of its limitations, see Section K, available as supplementary data at *Bioinformatics* online.

Beyond such methodological integration, certain challenges remain. First, the scarcity of high-resolution complex structures necessitates the use of structure prediction tools such as AlphaFold3 (Abramson *et al.* 2024). Second, reliance on tools like FoldX incurs computational burden at scale, highlighting the need for more efficient alternatives. Third, the current framework adopts a static-structure assumption via ProteinMPNN (*T* = 1.0), overlooking the flexibility of dynamic regions like antibody CDR loops. Antibody-specific models such as AbMPNN (Dreyer *et al.* 2023) offer a promising alternative for future work. Addressing these challenges would enhance the efficiency and scalability of the proposed *in silico* protein engineering pipeline, advancing biomolecular design and broader applications in disease therapy and biomedical research.

## Supplementary Material

btag249_Supplementary_Data

## Data Availability

The SKEMPI v2.0 dataset was obtained from the official repository at https://life.bsc.es/pid/skempi2/. The dataset for the antibody CR6261 against H1N1 was sourced from AbBiBench (https://doi.org/10.48550/arXiv.2506.04235), the dataset for the antibody trastuzumab against HER2 was sourced from GearBind (https://doi.org/10.1038/s41467-024-51563-8), and the dataset for the SARS-CoV-2 RBD against hACE2 was sourced from DiffAffinity (https://doi.org/10.48550/arXiv.2310.19849). All processed datasets used for the experimental evaluation in this article are available on GitHub at https://github.com/ak422/USP-ddG.
