# Supplementary material for: USP-ddG: a unified structural paradigm with data efficacy and mixture-of-experts for predicting mutational effects on protein–protein interactions
Source: Bioinformatics. 2026 Jul 7;42(Suppl 1):btag249. doi: 10.1093/bioinformatics/btag249 (PMC13340161; doi:10.1093/bioinformatics/btag249)
Supplement: btag249_Supplementary_Data [file btag249_supplementary_data.pdf]

USP-ddG: A Unified Structural Paradigm with Data Efficacy and Mixture-of-Experts for Predicting Mutational Effects on Protein-Protein Interactions

Guanglei Yu[1,2,3], Xuehua Bi[3], Qichang Zhao[1,2], and Jianxin Wang[1,2(✉)]

[1] School of Computer Science and Engineering, Central South University, Changsha 410083, China
[2] Hunan Provincial Key Lab on Bioinformatics, Central South University, Changsha 410083, China
jxwang@mail.csu.edu.cn
[3] College of Medical Engineering and Technology, Xinjiang Medical University, Urumqi 830017, China

Supplementary Material

In Section A, we describe how to use inverse folding model to predict $\Delta\Delta G$. In Section B, we provide a detailed derivation of the binding free energy ΔG^{SS} under the static-structure hypothesis. In Section C, we provide a detailed description of the geometric encoder of improved CATH-ddG. The procedure for applying Gaussian noise are presented in Sections D. In Section E and Section F, we provide a detailed description of the seven metrics used to evaluate $\Delta\Delta G$ prediction performance and the parameter settings in our proposed USP-ddG model. In Section G, we provide the data splitting results for SKEMPI v2.0 according to CATH homologous superfamily [14]. In Section H, we discuss the experiments conducted mutations on the SARS-CoV-2 RBD to hACE2. In Section I, we report the computational efficiency analysis of USP-ddG. In Section J, we report the computational efficiency and scalability analysis of USP-ddG for large-scale virtual screening. In Section K, we report the error analysis of performance limitations of USP-ddG.

A $\Delta\Delta G$ Prediction Using Inverse Folding

From the definition of ΔG, previous work such as BA-DDG [7] interprets Gibbs free energy G_{bnd} and G_{unbnd} as the conditional probabilities of the complex structure being in the bound conformation $\mathcal{X}_{\text{bnd}}$ or unbound conformation $\mathcal{X}_{\text{unbnd}}$, respectively, given its sequence $\mathcal{S}_{\mathcal{AB}}$. Thus, the binding free energy ΔG is defined by the Boltzmann distribution [1]:

$$
\begin{aligned}
G_{\text{bnd}} &= -k_B T \cdot \log p(\mathcal{X}_{\text{bnd}} \mid \mathcal{S}_{\mathcal{AB}}), && (1)\\
G_{\text{unbnd}} &= -k_B T \cdot \log p(\mathcal{X}_{\text{unbnd}} \mid \mathcal{S}_{\mathcal{AB}}), && (2)\\
\Delta G &= G_{\text{bnd}} - G_{\text{unbnd}} \\
&= -k_B T \cdot \left(\log p(\mathcal{X}_{\text{bnd}} \mid \mathcal{S}_{\mathcal{AB}}) - \log p(\mathcal{X}_{\text{unbnd}} \mid \mathcal{S}_{\mathcal{AB}})\right), && (3)
\end{aligned}
$$

where k_B is the Boltzmann constant and T is the thermodynamic temperature. Subsequently, applying Bayes' theorem into Eq. (3), this simplifies to:

$$
\begin{aligned}
\Delta G &= -\,k_B T \cdot \Big(\frac{\log p(\mathcal{S}_{\mathcal{AB}} \mid \mathcal{X}_{\text{bnd}}) \cdot p(\mathcal{X}_{\text{bnd}})}{p(\mathcal{S}_{\mathcal{AB}})} - \frac{\log p(\mathcal{S}_{\mathcal{AB}} \mid \mathcal{X}_{\text{unbnd}}) \cdot p(\mathcal{X}_{\text{unbnd}})}{p(\mathcal{S}_{\mathcal{AB}})}\Big), && (4)\\
&= -\,k_B T \cdot \log \frac{p(\mathcal{S}_{\mathcal{AB}} \mid \mathcal{X}_{\text{bnd}}) \cdot p(\mathcal{X}_{\text{bnd}})}{p(\mathcal{S}_{\mathcal{AB}} \mid \mathcal{X}_{\text{unbnd}}) \cdot p(\mathcal{X}_{\text{unbnd}})} && (5)
\end{aligned}
$$

Therefore, $\Delta\Delta G$ can be simplified via the thermodynamic cycle to:

$$
\begin{aligned}
\Delta\Delta G &= -\,k_B T \cdot \Big(\log \frac{p(\mathcal{S}^{\text{mut}}_{\mathcal{AB}} \mid \mathcal{X}^{\text{mut}}_{\text{bnd}}) \cdot p(\mathcal{X}^{\text{mut}}_{\text{bnd}})}{p(\mathcal{S}^{\text{mut}}_{\mathcal{AB}} \mid \mathcal{X}^{\text{mut}}_{\text{unbnd}}) \cdot p(\mathcal{X}^{\text{mut}}_{\text{unbnd}})} - \log \frac{p(\mathcal{S}^{\text{wt}}_{\mathcal{AB}} \mid \mathcal{X}^{\text{wt}}_{\text{bnd}}) \cdot p(\mathcal{X}^{\text{wt}}_{\text{bnd}})}{p(\mathcal{S}^{\text{wt}}_{\mathcal{AB}} \mid \mathcal{X}^{\text{wt}}_{\text{unbnd}}) \cdot p(\mathcal{X}^{\text{wt}}_{\text{unbnd}})}\Big), && (6)\\
&= -\,k_B T \cdot \Big(\log \frac{p(\mathcal{S}^{\text{mut}}_{\mathcal{AB}} \mid \mathcal{X}^{\text{mut}}_{\text{bnd}}) \cdot p(\mathcal{X}^{\text{mut}}_{\text{bnd}})}{p(\mathcal{S}^{\text{mut}}_{\mathcal{AB}} \mid \mathcal{X}^{\text{mut}}_{\text{unbnd}}) \cdot p(\mathcal{X}^{\text{wt}}_{\text{bnd}})} - \log \frac{p(\mathcal{S}^{\text{wt}}_{\mathcal{AB}} \mid \mathcal{X}^{\text{mut}}_{\text{unbnd}}) \cdot p(\mathcal{X}^{\text{mut}}_{\text{unbnd}})}{p(\mathcal{S}^{\text{wt}}_{\mathcal{AB}} \mid \mathcal{X}^{\text{mut}}_{\text{unbnd}}) \cdot p(\mathcal{X}^{\text{wt}}_{\text{unbnd}})}\Big). && (7)
\end{aligned}
$$

In addition, under the assumption that the protein backbone structure remains unchanged before and after mutation, this is mathematically equivalent to the following equalities:

$$\mathcal{X}^{\text{mut}}_{\text{bnd}} = \mathcal{X}^{\text{wt}}_{\text{bnd}}, \quad \mathcal{X}^{\text{mut}}_{\text{unbnd}} = \mathcal{X}^{\text{wt}}_{\text{unbnd}}, \tag{8}$$

$$\implies p(\mathcal{X}^{\text{mut}}_{\text{bnd}}) = p(\mathcal{X}^{\text{wt}}_{\text{bnd}}), \quad p(\mathcal{X}^{\text{mut}}_{\text{unbnd}}) = p(\mathcal{X}^{\text{wt}}_{\text{unbnd}}). \tag{9}$$

Then, we can eliminate the probability $\frac{p(\mathcal{X}^{\text{mut}}_{\text{bnd}})}{p(\mathcal{X}^{\text{wt}}_{\text{bnd}})}$ and $\frac{p(\mathcal{X}^{\text{mut}}_{\text{unbnd}})}{p(\mathcal{X}^{\text{wt}}_{\text{unbnd}})}$ in Eq. (7), and express $\Delta\Delta G$ as follows:

$$\Delta\Delta G = -\, k_B T \cdot \left(\log \frac{p(\mathcal{S}^{\text{mut}}_{\mathcal{AB}} \mid \mathcal{X}^{\text{mut}}_{\text{bnd}})}{p(\mathcal{S}^{\text{mut}}_{\mathcal{AB}} \mid \mathcal{X}^{\text{mut}}_{\text{unbnd}})} - \log \frac{p(\mathcal{S}^{\text{wt}}_{\mathcal{AB}} \mid \mathcal{X}^{\text{wt}}_{\text{bnd}})}{p(\mathcal{S}^{\text{wt}}_{\mathcal{AB}} \mid \mathcal{X}^{\text{wt}}_{\text{unbnd}})} \right), \tag{10}$$

where $p(\mathcal{S}^{\text{mut}}_{\mathcal{AB}} \mid \mathcal{X}^{\text{mut}}_{\text{unbnd}})$ and $p(\mathcal{S}^{\text{wt}}_{\mathcal{AB}} \mid \mathcal{X}^{\text{wt}}_{\text{unbnd}})$, the conditional probabilities of the mutant and wild-type unbound states, are approximated under the assumption of independence between the two monomers as follows:

$$p(\mathcal{S}^{\text{mut}}_{\mathcal{AB}} \mid \mathcal{X}^{\text{mut}}_{\text{unbnd}}) \approx p(\mathcal{S}^{\text{mut}}_{\mathcal{A}} \mid \mathcal{X}^{\text{mut}}_{\mathcal{A}}) \cdot p(\mathcal{S}^{\text{mut}}_{\mathcal{B}} \mid \mathcal{X}^{\text{mut}}_{\mathcal{B}}), \tag{11}$$

$$p(\mathcal{S}^{\text{wt}}_{\mathcal{AB}} \mid \mathcal{X}^{\text{wt}}_{\text{unbnd}}) \approx p(\mathcal{S}^{\text{wt}}_{\mathcal{A}} \mid \mathcal{X}^{\text{wt}}_{\mathcal{A}}) \cdot p(\mathcal{S}^{\text{wt}}_{\mathcal{B}} \mid \mathcal{X}^{\text{wt}}_{\mathcal{B}}). \tag{12}$$

Under these assumptions, inverse folding models such as ProteinMPNN compute the negative log-likelihood of a protein sequence conditioned on its backbone structure, yielding the following approximations in Eq. (10):

$$\Delta G^{\text{mut}}_{\text{ProteinMPNN}} = -k_B T \cdot \Big(\log p(\mathcal{S}^{\text{mut}}_{\mathcal{AB}} \,|\, \mathcal{X}^{\text{mut}}_{\text{bnd}}) - \log p(\mathcal{S}^{\text{mut}}_{\mathcal{A}} \,|\, \mathcal{X}^{\text{mut}}_{\mathcal{A}}) - \log p(\mathcal{S}^{\text{mut}}_{\mathcal{B}} \,|\, \mathcal{X}^{\text{mut}}_{\mathcal{B}}) \Big), \tag{13}$$

$$\Delta G^{\text{wt}}_{\text{ProteinMPNN}} = -k_B T \cdot \Big(\log p(\mathcal{S}^{\text{wt}}_{\mathcal{AB}} \,|\, \mathcal{X}^{\text{wt}}_{\text{bnd}}) - \log p(\mathcal{S}^{\text{wt}}_{\mathcal{A}} \,|\, \mathcal{X}^{\text{wt}}_{\mathcal{A}}) - \log p(\mathcal{S}^{\text{wt}}_{\mathcal{B}} \,|\, \mathcal{X}^{\text{wt}}_{\mathcal{B}}) \Big). \tag{14}$$

Consequently, all conditional probabilities in Eq. (10) can be directly computed from the negative log-likelihood scores generated by ProteinMPNN. Thus, the change in binding free energy is defined as:

$$\begin{aligned} \Delta\Delta G &\triangleq \Delta\Delta G_{\text{ProteinMPNN}} \\ &= \Delta G^{\text{mut}}_{\text{ProteinMPNN}} - \Delta G^{\text{wt}}_{\text{ProteinMPNN}}. \end{aligned} \tag{15}$$

B Static-structure Assumption

To quantify the binding free energy under the static-structure and conformational-ensemble hypotheses, we first define the corresponding structural representations. Let $\mathcal{X}$ denotes the protein structure, which is described differently under each hypothesis:

$$\mathcal{X}^{\text{SS}} = \{\mathcal{X}^{\text{SS}}_{\text{BB}}, \mathcal{X}^{\text{SS}}_{\text{SC}}\}, \qquad \mathcal{X}^{\text{CE}} = \{\mathcal{X}^{\text{CE}}_{\text{BB}}, \mathcal{X}^{\text{CE}}_{\text{SC}}\}, \tag{16}$$

where $\mathcal{X}^{\text{SS}}$ corresponds to the protein structure under the static-structure hypothesis, defined by a unique backbone $\mathcal{X}^{\text{SS}}_{\text{BB}}$ and side-chain $\mathcal{X}^{\text{SS}}_{\text{SC}}$. In contrast, $\mathcal{X}^{\text{CE}}$ represents the structure under the conformational-ensemble hypothesis, characterized by a set of backbones $\mathcal{X}^{\text{CE}}_{\text{BB}}$ and side-chains $\mathcal{X}^{\text{CE}}_{\text{SC}}$.

Under the static-structure hypothesis, the binding free energy ΔG^{SS} can be derived from the Boltzmann distribution [1], and expressed in terms of the probabilities of the bound state $p^{\text{SS}}_{\text{bnd}}$ and the unbound state $p^{\text{SS}}_{\text{unbnd}}$. Unlike the conditional probability formulation $P(\mathcal{X} \mid \mathcal{S})$ commonly adopted in prior protein inverse folding studies, our approach targets the joint probability $P(\mathcal{X}, \mathcal{S}) \propto e^{-k_\beta T \cdot \Delta G^{\text{SS}}}$, allowing for consistent and unified modeling of both sequence and structure under mutation across different protein conformational assumptions. Thus, the binding free energy ΔG^{SS} under the static-structure hypothesis is defined as:

$$\begin{aligned} \Delta G^{\text{SS}} &= G^{\text{SS}}_{\text{bnd}} - G^{\text{SS}}_{\text{unbnd}} \\ &= -k_B T \cdot \left(\log p^{\text{SS}}_{\text{bnd}} - \log p^{\text{SS}}_{\text{unbnd}}\right) \\ &= -k_B T \cdot \Big(\log p(\mathcal{X}^{\text{SS}}_{\text{bnd}}, \mathcal{S}_{\mathcal{AB}}) - \log p(\mathcal{X}^{\text{SS}}_{\text{unbnd}}, \mathcal{S}_{\mathcal{AB}}) \Big). \end{aligned} \tag{17}$$

Applying the product rule of probability, $p(\mathcal{X}, \mathcal{S}) = p(\mathcal{S} \mid \mathcal{X}) \cdot p(\mathcal{X})$, we can derive the following:

$$\log p(\mathcal{X}^{\mathrm{SS}}_{\mathrm{bnd}}, \mathcal{S}_{\mathcal{AB}}) = \log p(\mathcal{S}_{\mathcal{AB}} \mid \mathcal{X}^{\mathrm{SS}}_{\mathrm{bnd}}) \cdot p(\mathcal{X}^{\mathrm{SS}}_{\mathrm{bnd}}), \tag{18}$$

$$\log p(\mathcal{X}^{\mathrm{SS}}_{\mathrm{unbnd}}, \mathcal{S}_{\mathcal{AB}}) = \log p(\mathcal{S}_{\mathcal{AB}} \mid \mathcal{X}^{\mathrm{SS}}_{\mathrm{unbnd}}) \cdot p(\mathcal{X}^{\mathrm{SS}}_{\mathrm{unbnd}}). \tag{19}$$

Then, the joint probability of the bound and unbound structure given the sequence and structure can be decomposed as:

$$\Delta G^{\mathrm{SS}} = -k_B T \cdot \Big(\log p(\mathcal{S}_{\mathcal{AB}} \mid \mathcal{X}^{\mathrm{SS}}_{\mathrm{bnd}}) \cdot p(\mathcal{X}^{\mathrm{SS}}_{\mathrm{bnd}}) - \log p(\mathcal{S}_{\mathcal{AB}} \mid \mathcal{X}^{\mathrm{SS}}_{\mathrm{unbnd}}) \cdot p(\mathcal{X}^{\mathrm{SS}}_{\mathrm{unbnd}}) \Big) \tag{20}$$

After that, we get:

$$\Delta G^{\mathrm{SS}} = -k_B T \cdot \log \frac{p(\mathcal{S}_{\mathcal{AB}} \mid \mathcal{X}^{\mathrm{SS}}_{\mathrm{bnd}}) \cdot p(\mathcal{X}^{\mathrm{SS}}_{\mathrm{bnd}})}{p(\mathcal{S}_{\mathcal{AB}} \mid \mathcal{X}^{\mathrm{SS}}_{\mathrm{unbnd}}) \cdot p(\mathcal{X}^{\mathrm{SS}}_{\mathrm{unbnd}})}, \tag{21}$$

$$= -k_B T \cdot \log \frac{p(\mathcal{S}_{\mathcal{AB}} \mid \mathcal{X}^{\mathrm{SS}}_{\mathrm{bnd}})}{p(\mathcal{S}_{\mathcal{AB}} \mid \mathcal{X}^{\mathrm{SS}}_{\mathrm{unbnd}})} - k_B T \cdot \log \frac{p(\mathcal{X}^{\mathrm{SS}}_{\mathrm{bnd}})}{p(\mathcal{X}^{\mathrm{SS}}_{\mathrm{unbnd}})}. \tag{22}$$

Under the static-structure assumption, we assume that the two monomers are structurally independent in the unbound state. Consequently, $p(\mathcal{S}_{\mathcal{AB}} \,|\, \mathcal{X}^{\mathrm{SS}}_{\mathrm{unbnd}})$ can be approximately factorized into product of the two monomers:

$$p(\mathcal{S}_{\mathcal{AB}} \,|\, \mathcal{X}^{\mathrm{SS}}_{\mathrm{unbnd}}) \approx p(\mathcal{S}_{\mathcal{A}} \,|\, \mathcal{X}^{\mathrm{SS}}_{\mathcal{A}}) \cdot p(\mathcal{S}_{\mathcal{B}} \,|\, \mathcal{X}^{\mathrm{SS}}_{\mathcal{B}}). \tag{23}$$

Furthermore, leveraging the common assumption in protein sequence design that the sequence is largely determined by the backbone structure, we approximate:

$$\log p(\mathcal{S}_{\mathcal{AB}} \mid \mathcal{X}^{\mathrm{SS}}_{\mathrm{bnd}}) = \log p(\mathcal{S}_{\mathcal{AB}} \mid \{\mathcal{X}^{\mathrm{SS}}_{\mathrm{bnd,BB}}, \mathcal{X}^{\mathrm{SS}}_{\mathrm{bnd,SC}}\}) \approx \log p(\mathcal{S}_{\mathcal{AB}} \mid \mathcal{X}^{\mathrm{SS}}_{\mathrm{bnd,BB}}), \tag{24}$$

$$\log p(\mathcal{S}_{\mathcal{A}} \mid \mathcal{X}^{\mathrm{SS}}_{\mathcal{A}}) = \log p(\mathcal{S}_{\mathcal{A}} \mid \{\mathcal{X}^{\mathrm{SS}}_{\mathcal{A},\mathrm{BB}}, \mathcal{X}^{\mathrm{SS}}_{\mathcal{A},\mathrm{SC}}\}) \approx \log p(\mathcal{S}_{\mathcal{A}} \mid \mathcal{X}^{\mathrm{SS}}_{\mathcal{A},\mathrm{BB}}), \tag{25}$$

$$\log p(\mathcal{S}_{\mathcal{B}} \mid \mathcal{X}^{\mathrm{SS}}_{\mathcal{B}}) = \log p(\mathcal{S}_{\mathcal{B}} \mid \{\mathcal{X}^{\mathrm{SS}}_{\mathcal{B},\mathrm{BB}}, \mathcal{X}^{\mathrm{SS}}_{\mathcal{B},\mathrm{SC}}\}) \approx \log p(\mathcal{S}_{\mathcal{B}} \mid \mathcal{X}^{\mathrm{SS}}_{\mathcal{B},\mathrm{BB}}). \tag{26}$$

Thus, the first term in Eq. (22) can be estimated using the pre-trained inverse folding model ProteinMPNN, and the corresponding negative log-likelihood scores are collectively denoted as:

$$\Delta G_{\mathrm{ProteinMPNN}} = -k_B T \cdot \Big(\log p(\mathcal{S}_{\mathcal{AB}} \,|\, \mathcal{X}^{\mathrm{SS}}_{\mathrm{bnd}}) - \log p(\mathcal{S}_{\mathcal{A}} \,|\, \mathcal{X}^{\mathrm{SS}}_{\mathcal{A}}) - \log p(\mathcal{S}_{\mathcal{B}} \,|\, \mathcal{X}^{\mathrm{SS}}_{\mathcal{B}}) \Big). \tag{27}$$

And the second term in Eq. (22) serves as a conformational energy term of the complex under the static-structure assumption. Prior work EBM-DDG approximated this term using a score-based energy model, DSMBind [8], which leverages denoising score matching to sample low energy mutant structures and compute the energy difference. In contrast, our method explicitly models the change in side-chain packing energetics upon mutation using the empirical force field FoldX. This is achieved using its "Interaction Energy" term, denoted as $\Delta G_{\mathrm{FoldX}}$:

$$\begin{aligned} \Delta G_{\mathrm{FoldX}} &= -k_B T \cdot \log \frac{p(\mathcal{X}^{\mathrm{SS}}_{\mathrm{bnd}})}{p(\mathcal{X}^{\mathrm{SS}}_{\mathrm{unbnd}})} \\ &= -k_B T \cdot \log \frac{p(\{\mathcal{X}^{\mathrm{SS}}_{\mathrm{bnd,BB}}, \mathcal{X}^{\mathrm{SS}}_{\mathrm{bnd,SC}}\})}{p(\{\mathcal{X}^{\mathrm{SS}}_{\mathrm{unbnd,BB}}, \mathcal{X}^{\mathrm{SS}}_{\mathrm{unbnd,SC}}\})}. \end{aligned} \tag{28}$$

Thus, under the aforementioned static-structure assumption, the changes in binding free energy $\Delta\Delta G^{\mathrm{SS}}$ is derived as follows:

$$\begin{aligned} \Delta\Delta G^{\mathrm{SS}} &= \Delta G^{\mathrm{SS,mut}} - \Delta G^{\mathrm{SS,wt}} \\ &= (\Delta G^{\mathrm{mut}}_{\mathrm{ProteinMPNN}} + \Delta G^{\mathrm{mut}}_{\mathrm{FoldX}}) - (\Delta G^{\mathrm{wt}}_{\mathrm{ProteinMPNN}} + \Delta G^{\mathrm{wt}}_{\mathrm{FoldX}}) \\ &= (\Delta G^{\mathrm{mut}}_{\mathrm{ProteinMPNN}} - \Delta G^{\mathrm{wt}}_{\mathrm{ProteinMPNN}}) + (\Delta G^{\mathrm{mut}}_{\mathrm{FoldX}} - \Delta G^{\mathrm{wt}}_{\mathrm{FoldX}}) \\ &= \Delta\Delta G_{\mathrm{ProteinMPNN}} + \Delta\Delta G_{\mathrm{FoldX}}. \end{aligned} \tag{29}$$

C Geometric encoder of improved CATH-ddG

The geometric encoder of improved CATH-ddG consists of two components: a spatial geometric encoder followed by a sequential geometric encoder. In the spatial geometric encoder, we iterate over residues within the input patch and perform 3D spatial cropping under the constraints $k_1 = 20$ and $k_2 = 7$, resulting in a spatial graph with $k_1 + k_2$ edges per residue. In the sequential geometric encoder, residues are selected according to the constraint $k_3 = 3$, forming a sequential graph with k_3 edges per residue.

The sequential geometric encoder shares the same architecture as the spatial geometric encoder. The forward process of the overall geometric structure encoder at the l-th layer is defined as follows:

$$\mathbf{m}_{ij}^{(l)} = \texttt{MLP}\Big(\texttt{Concat}\big(\texttt{LN}(\mathbf{h}_i^{(l-1)}), \texttt{LN}(\mathbf{h}_j^{(l-1)}), \mathbf{e}_{ij}^{(l-1)}\big)\Big), \tag{30}$$

$$\mathbf{h}_i^{(l)} = \mathbf{h}_i^{(l-1)} + \mathbf{M}^{(l)} \odot \Big(\alpha^{(l)} \frac{\sum_{j\in N_{(i,k)}} \mathbf{m}_{ij}^{(l)}}{\lambda_i}\Big), \tag{31}$$

$$\mathbf{h}_{\texttt{FFN}} = \texttt{FFN}(\texttt{LN}(\mathbf{h}_i^{(l)})), \tag{32}$$

$$\mathbf{h}_{\texttt{MoE}} = \texttt{MoE}(\texttt{LN}(\mathbf{h}_i^{(l)})), \tag{33}$$

$$\mathbf{h}_i^{(l)} = \mathbf{h}_i^{(l)} + \mathbf{M}^{(l)} \odot \Big(\alpha^{(l)} \cdot \texttt{Concat}\big(\mathbf{h}_{\texttt{FFN}}, \mathbf{h}_{\texttt{MoE}}\big)\Big), \tag{34}$$

$$\mathbf{e}_{ij}^{(l)} = \texttt{MLP}\Big(\texttt{Concat}\big(\mathbf{h}_i^{(l)}, \mathbf{h}_j^{(l)}, \texttt{LN}(\mathbf{e}_{ij}^{(l-1)})\big)\Big), \tag{35}$$

$$\mathbf{e}_{ij}^{(l)} = \mathbf{e}_{ij}^{(l-1)} + \mathbf{M}^{(l)} \odot (\mathbf{e}_{ij}^{(l)}), \tag{36}$$

where $\texttt{MLP}(\cdot)$ denotes a multi-layer perceptron with $\texttt{ReLU}(\cdot)$ [5] as the activation function, and $\texttt{LN}(\cdot)$ represents layer normalization without bias [13]. $N_{(i,k)}$ denotes the set of k-nearest neighbors of node i, $\texttt{FFN}(\cdot)$ is a feed-forward network, and $\texttt{MoE}(\cdot)$ indicates the Mixture-of-Experts module. $\alpha^{(l)}$ is a learnable scalar used in ReZero [2], $\lambda_i = |N_{(i,k)}|$, and the dropout mask $\mathbf{M}^{(l)} \sim \text{Bernoulli}(p = 0.1)$). The node inputs to the spatial and sequential geometric encoders are $\mathbf{h}_i^{(0)}$ and $\mathbf{h}_i^{\text{spatial}}$, respectively; that is, the sequential encoder takes the outputs of the spatial encoder as its inputs. Notably, $\mathbf{x} = \{\mathbf{h}_i^{(l)}\}_{i=1}^{L_{\text{patch}}}$ is the input tokens of $\texttt{MoE}(\cdot)$.

Finally, we take the output $\mathbf{h}_i^{\text{spatial}}$ and $\mathbf{h}_i^{\text{sequential}}$ as node representations, and $\mathbf{e}_{ij}^{\text{spatial}}$ and $\mathbf{e}_{ij}^{\text{sequential}}$ as edge representations, respectively, which are used as inputs to the fusion layer. Subsequently, the fusion layer is given by:

$$\mathbf{h}_i^{\text{enc}} = \texttt{Concat}(\mathbf{h}_i^{\text{spatial}}, \mathbf{h}_i^{\text{sequential}})\mathbf{W}, \quad \mathbf{e}_{ij}^{\text{enc}} = \texttt{Merge}(\mathbf{e}_{ij}^{\text{spatial}}, \mathbf{e}_{ij}^{\text{sequential}}), \tag{37}$$

$$\mathbf{m}_{ij} = \texttt{MLP}\big(\texttt{Concat}(\mathbf{h}_i^{(0)}, \mathbf{h}_j^{(0)}, \mathbf{h}_i^{\text{enc}}, \mathbf{h}_j^{\text{enc}}, \mathbf{e}_{ij}^{\text{enc}})\big), \tag{38}$$

$$\mathbf{h}_i^{\text{fusion}} = \texttt{LN}\big(\mathbf{h}_i^{\text{enc}} + \mathbf{M} \odot (\frac{\sum_{j\in N_{(i,k)}} \mathbf{m}_{ij}}{\lambda_i})\big), \tag{39}$$

where $\texttt{Concat}(\cdot)$ denotes concatenation across feature dimensions, $\texttt{Merge}(\cdot)$ represents the merging operation across edge channels, and $\mathbf{W} \in \mathbb{R}^{2d_{\text{model}} \times d_{\text{model}}}$is a learnable parameter matrix for node fusion. The outputs of the fusion layer are subsequently normalized by the residue CentralityNorm layer, yielding the final node representations $\mathbf{h}_i$ as follows:

$$\hat{c_i} = \frac{\sum_{j\in N_{(i,k)}} c_{ij} + w}{||\sum_{j\in N_{(i,k)}} c_{ij} + w||_2}, \tag{40}$$

$$\mathbf{h}_i = \hat{c_i} \cdot \mathbf{h}_i^{\text{fusion}}, \tag{41}$$

where $\hat{c_i} \in [0, 1]$ is l_2 normalization for residue r_i, c_{ij} is the residue centrality of r_i defined in [10], w is a learnable scalar parameter, $i \in \{L_{\text{patch}}\} \triangleq \{1, 2, \ldots, L_{\text{patch}}\}$, $L_{\text{patch}} = 256$ is the length of cropped residue patch.

The design of the geometric SE(3)-invariant function mapping transforms the protein 3D coordinates $\boldsymbol{x}_i$ into fundamental invariant scalar representations for each residue r_i. These representations include edge features $\boldsymbol{e}_{ij}$, which are defined in the local coordinate frame $\boldsymbol{O}_i = [\boldsymbol{b}_i \ \ \boldsymbol{n}_i \ \ \boldsymbol{b}_i \times \boldsymbol{n}_i]$ associated with residue

r_i, and are decomposed into distance, direction, and orientation components, as detailed in [6].

$$\boldsymbol{e}_{ij}^{\text{distance}} = \boldsymbol{\Phi}\left(||\boldsymbol{x}_j - \boldsymbol{x}_i||\right), \tag{42}$$

$$\boldsymbol{e}_{ij}^{\text{direction}} = \boldsymbol{O}_i^T \frac{\boldsymbol{x}_j - \boldsymbol{x}_i}{||\boldsymbol{x}_j - \boldsymbol{x}_i||}, \tag{43}$$

$$\boldsymbol{e}_{ij}^{\text{orientation}} = \mathbf{q}(\boldsymbol{O}_i^T \boldsymbol{O}_j), \tag{44}$$

To construct SE(3)-invariant node features, we compute the backbone dihedral angles (ϕ_i, ψ_i) and the side-chain dihedral angles $(\chi_i^{(1)}, \chi_i^{(2)}, \chi_i^{(3)}, \chi_i^{(4)})$ for each residue r_i. These angular features are then embedded into a toroidal space using sine and cosine transformations, i.e., $\{\sin(\cdot), \cos(\cdot)\} \times (\phi_i, \psi_i, \chi_i^{(1)}, \chi_i^{(2)}, \chi_i^{(3)}, \chi_i^{(4)})$.

D Data augmentation

Data augmentation is a widely adopted training technique that improves model robustness by increasing the scale and diversity of training data without collecting additional samples. In the improved CATH-ddG, we employ data augmentation from three complementary perspectives to computationally approximate the conformational ensemble of the protein as input.

First, training with coordinate noise has been shown to be an effective strategy in protein design [3] and in 3D molecular pre-training for coordinate denoising [4]. Building on these insights, we leverage a hybrid noise strategy that applies two-stage Gaussian perturbations with different variances to the atomic coordinates of the rigid backbone and the more flexible side-chains. Specifically, in the first step, the rigid backbone and flexible side-chain components are perturbed using Gaussian noise with standard deviations of $\delta = 0.10$ Å and $\delta = 0.20$ Å, respectively, to generate perturbed dihedral features at the node level. In the second step, the original atomic coordinates are further perturbed using the same noise scheme, resulting in perturbed distance, direction, and orientation features for both nodes and edges. Through this two-stage process, the hybrid noise strategy introduces differentiated perturbations that impose mild anisotropic constraints on the local conformational variations of proteins.

Second, certain side-chain dihedral angles exhibit symmetric invariance, meaning that flipping these angles does not alter the overall 3D structure [10]. This includes $\chi^{(2)}$ of aspartic acid (ASP), $\chi^{(3)}$ of glutamic acid (GLU), ($\chi^{(2)}$ of phenylalanine (PHE), and $\chi^{(2)}$ of tyrosine (TYR). We exploit this property as a data augmentation strategy to increase structural diversity. Specifically, the corresponding side-chain dihedral angles are rotated by 180° with a uniform probability of $p = 0.2$.

Finally, the chains within each protein complex are randomly shuffled during training to mitigate ordering bias.

E Evaluation Metrics

We employed a comprehensive set of seven metrics to evaluate $\Delta\Delta G$ prediction performance. These included five overall metrics: (1) Pearson correlation coefficient; (2) Spearman's rank correlation coefficient; (3) Root Mean Squared Error (RMSE); (4) Mean Absolute Error (MAE); and (5) AUROC, where mutations were classified based on the sign of ground-truth $\Delta\Delta G$ values. Given the practical importance of correlation within specific PPIs, we also reported two additional PPI-wise metrics. Mutations were grouped by PPI, and the average Pearson and Spearman correlation coefficients across PPIs were reported as Per-PPI correlation metrics.

F Parameter Settings

We implemented USP-ddG with PyTorch, setting the model dimension to $d_{\text{model}} = 128$ and using a batch size of 12 on a single NVIDIA GeForce RTX 2080 Ti GPU. The Mixture-of-Experts (MoE) layer was configured with 12 experts, activating the top-2 experts for each input token via a LoRA-based MoE-Adapter of rank $r = 64$; notably, we did not employ a load-balancing auxiliary loss. Model parameters were optimized using the Adam algorithm with default parameters ($\beta_1 = 0.9, \beta_2 = 0.999$) and a cyclical cosine annealing scheduler.

The four learnable loss weights $\{\lambda_i\}$ in Eq. (12) were initialized to 1.0 and converged to $\lambda_1 = 0.5344$ for the ProteinMPNN-based inverse folding task, $\lambda_2 = 2.3505$ for FoldX-based energy function task, $\lambda_3 = 0.9657$ for CATH-ddG supervised learning task, and $\lambda_4 = 3.0182$ for CATH self-supervised learning task. Regarding the CATH-guided Folding Ordering (CFO) curriculum learning, training data are organized into difficulty stages based on CATH classes, progressing from simpler to more complex folds. All samples within each stage are processed sequentially before moving to the next, and this stage-wise progression is repeated across epochs, ensuring a deterministic and reproducible training schedule. Following the CATH-ddG protocol, we augmented data by adding hybrid Gaussian noise to atomic coordinates with standard deviations of $\delta = 0.10$ Å for backbone atoms and $\delta = 0.20$ Å for side-chain atoms, and cropped each structure into patches of $L_{\text{patch}} = 256$ residues by selecting mutation sites as anchors and their 255 nearest neighbors based on C_β-C_β distances.

G Dataset

The SKEMPI v2.0 dataset is partitioned into a training set and a hold-out CATH test set based on CATH homologous superfamilies for protein complexes [14], with putative domain superfamilies for unannotated chains assigned through Foldseek structural similarity matching [12], as illustrated in Figure S1. To confirm that no data leakage exists between the training set and the hold-out CATH test set, the sequence and structural similarities between the hold-out CATH test set and the training set have been evaluated. The sequence identity is maintained below 30%, and no mutation entry in the hold-out CATH test set is classified as an easy mutation (maximum TM-score $\geq$ 0.6). In this partition, a mutation is defined as hard if its maximum TM-score is below 0.6 when compared to the training set, and as easy otherwise.

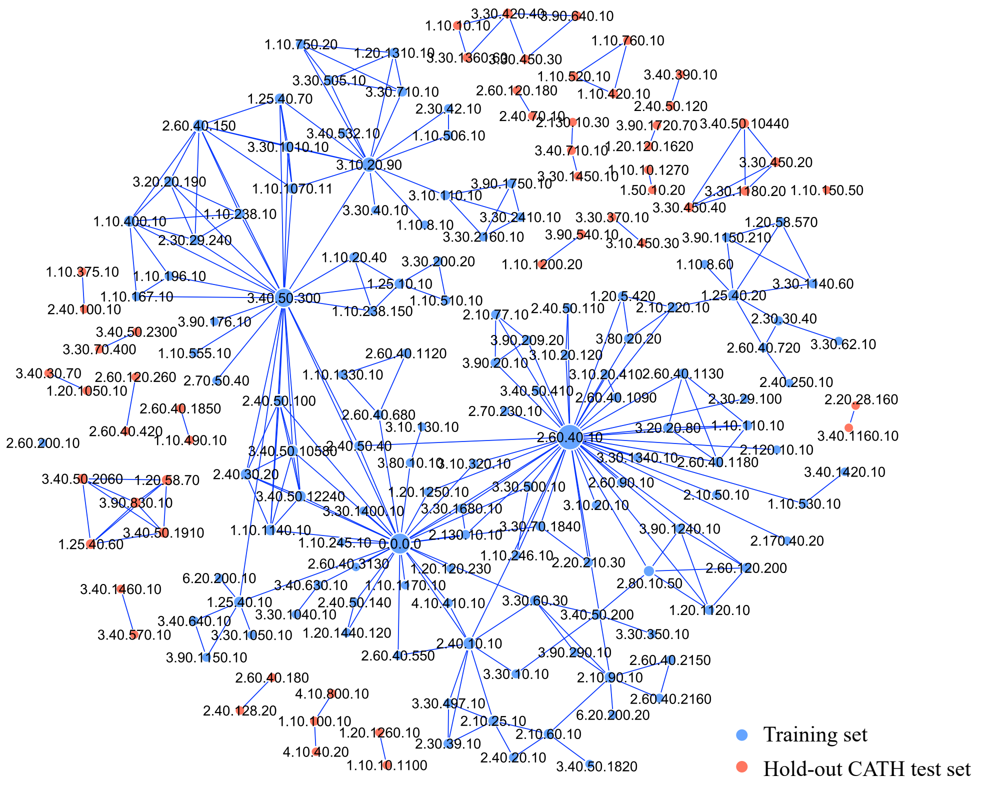

Fig. S1: Overview of data splitting for SKEMPI v2.0 according to CATH homologous superfamily.

H Evaluation on the SARS-CoV-2 RBD to hACE2

The evolution of the SARS-CoV-2 spike receptor-binding domain (RBD) is shaped by epistasis, leading to accumulated substitutions that enhance ACE2 binding affinity and enable escape from antibody recognition. To comprehensively quantify how epistasis alters mutational effects, [11] performed deep mutational scans (DMS) on the ancestral Wuhan-Hu-1 RBDs of all single-point mutations. Following [9], we benchmark predictions against 285 single-point mutations across 15 critical RBD sites. As shown in Table S1, USP-ddG achieves a PearsonR of 0.598 between the experimental and predicted $\Delta\Delta G$, markedly surpassing all other baseline methods.

Table S1: Evaluation of the binding affinity between SARS-CoV-2 RBD and hACE2[a].

Method	FoldX[b]	RDE-Network[b]	CATH-ddG[b]	BA-DDG[b]	USP-ddG^{Random}	USP-ddG
PearsonR↑	0.385	0.403	0.579	0.473	0.572	**0.598**±0.059

[a] The **bold** value indicates the best result.
[b] Results are from released tools or source code.

I Computational efficiency analysis of USP-ddG

We report the computational requirements for training USP-ddG in terms of parameter count, floating-point operations (FLOPs), and wall-clock time. The model contains 8.66 M trainable parameters and performs 15.6 G FLOPs per forward pass. A full training run on the SKEMPI v2.0 dataset (with the hold-out CATH dataset as the test set) completes in approximately 5.5 hours on a single NVIDIA GeForce RTX 2080 Ti GPU, with graphics memory usage of 8.9 GB.

For the computational requirements during inference, we first count the sequence length distribution of protein complexes in the SKEMPI2 dataset, as shown in Figure S2. Among them, the shortest sequence length is 57, the longest sequence length is 3397, and the proportion of sequence lengths less than 1000 is 95%.

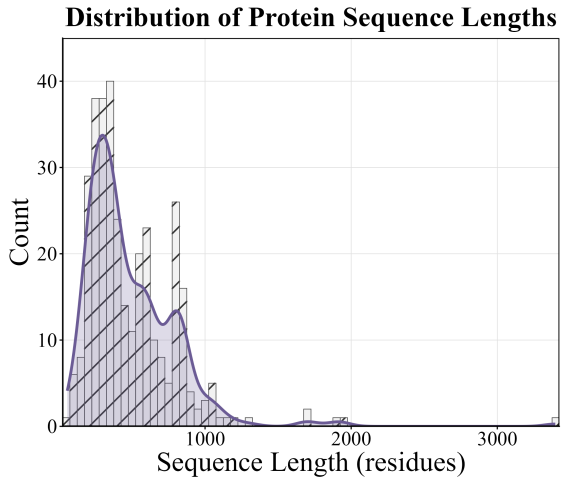

Fig. S2: Sequence length distribution of SKEMPI v2.0 dataset.

Second, we benchmark the time efficiency of flex ddG for both single-point and multiple mutations across proteins of short (57 amino acids), intermediate (1,015 amino acids), and long (3,397 amino acids) sequence lengths. All benchmarks are performed on a single core of an Intel Xeon Gold 6230R processor, hereinafter referred to as Xeon Gold 6230R. The default parameters used for flex ddG are provided in Listing S1. The corresponding computational efficiency analysis for flex ddG is detailed in Table S2.

```
        '-s %s' % input_pdb_path,
        '-parser:protocol', 'ddG-backrub.xml',
        '-parser:script_vars',
        'chainstomove=' + chains_to_move,
        'mutate_resfile_relpath=' + 'nataa_mutations.resfile',
        'number_backrub_trials=%d' % 35000,
        'max_minimization_iter=%d' % 5000,
        'abs_score_convergence_thresh=%.1f' % 1.0,
        'backrub_trajectory_stride=%d' % 7000,
        '-restore_talaris_behavior',
        '-in:file:fullatom',
        '-ignore_unrecognized_res',
        '-ignore_zero_occupancy false',
        '-ex1',
        '-ex2',
```

Listing S1: The default Rosetta Scripts protocol for flex ddG

Table S2: Computational efficiency analysis of flex ddG.

PDB ID	Length of the Sequences	Number of the Mutations	Total time (s)	Device
1KNE	57	1	671.82	Xeon Gold 6230R
1N8Z	1015	1	68,723.36	
3VR6	3397	1	288,363.88	
1KNE	57	5	687.79	
1N8Z	1015	5	73,004.53	
3VR6	3397	5	301,529.41	

Finally, to thoroughly assess the practical feasibility of USP-ddG for large-scale virtual screening, we conduct a computational cost analysis on three representative protein complexes, that is : PDB ID 1NKE (57 amino acids, the shortest), PDB ID 1N8Z (1,015 amino acids, corresponding to the 95.59% quantile of the sequence length distribution), and PDB ID 3VR6 (3,397 amino acids, the longest). All experiments are performed on a single core of a Xeon Gold 6230R processor for FoldX, and a single NVIDIA GeForce RTX 2080 Ti GPU (hereinafter referred to as RTX 2080 Ti) for USP-ddG. For each complex, we measured the wall-clock time for processing mutants containing 1, 2, and 5 mutation sites. A detailed breakdown of the computational efficiency, including the time spent on FoldX preprocessing, feature extraction, and model inference, is reported in Table S3.

J Computational Efficiency and Scalability Analysis for Large-Scale Virtual Screening

We select three representative protein complexes from the SKEMPI v2.0 dataset—1KNE (57 residues), 1N8Z (1015 residues), and 3VR6 (3397 residues)—to evaluate computational efficiency across different protein scales. As summarized in Table S3, the computational time increases with both protein length and mutation order. The measured times per mutant type for each protein are as follows: For the smallest protein, 1KNE, single-point mutations take 15.220 seconds, double mutants 27.451 seconds, and five-point mutants 35.770 seconds. For the medium-sized 1N8Z, times are 23.457 seconds, 36.153 seconds, and 87.360 seconds, respectively. For the largest complex, 3VR6, the corresponding times increase to 92.291 seconds, 100.211 seconds, and 172.345 seconds.

As shown in Table S4, the data reveal that single-point mutants exhibit a notably higher unit cost (23.46 seconds per site) compared to double mutants (18.077 seconds per site) and five-point mutants (17.472 seconds per site), indicating the presence of a fixed initialization overhead such as structure loading and

Table S3: Computational efficiency analysis of USP-ddG.

PDB ID	Length of the Sequences	Number of Mutations	Tools	Pipline	Wall-clock time (s)	
					Steps	All
1KNE	57	1	FoldX	Preprocessing	13.253	15.22
			USP-ddG	Feature extraction	1.686	
				Inference	0.281	
		2	FoldX	Preprocessing	23.625	25.487
			USP-ddG	Feature extraction	1.583	
				Inference	0.279	
		5	FoldX	Preprocessing	69.832	71.670
			USP-ddG	Feature extraction	1.567	
				Inference	0.271	
1N8Z	1015	1	FoldX	Preprocessing	20.318	23.457
			USP-ddG	Feature extraction	2.868	
				Inference	0.271	
		2	FoldX	Preprocessing	33.145	36.153
			USP-ddG	Feature extraction	2.726	
				Inference	0.282	
		5	FoldX	Preprocessing	84.516	87.360
			USP-ddG	Feature extraction	2.566	
				Inference	0.278	
3VR6	3397	1	FoldX	Preprocessing	84.940	92.291
			USP-ddG	Feature extraction	7.057	
				Inference	0.294	
		2	FoldX	Preprocessing	93.002	100.211
			USP-ddG	Feature extraction	6.912	
				Inference	0.297	
		5	FoldX	Preprocessing	164.497	172.345
			USP-ddG	Feature extraction	7.582	
				Inference	0.266	

Table S4: Per-mutation-site processing time derived from empirical measurements.

Mutant Type	Total Time (s)	Number of Mutation Sites	Time per Site (s)
Single-point	23.457	1	23.457
Double-point	36.153	2	18.077
Five-point	87.360	5	17.472

model warm-up. As the number of mutation sites increases, the per-site cost converges to approximately 17.472 seconds per site. For conservative estimation of large-scale screening costs, we adopt the average of double and five-point mutants, approximately 17.8 seconds per mutation site, as the baseline for multi-point screening scenarios, while a higher baseline of 23.5 seconds per site is used for tasks dominated by single-point mutations.

Based on these unit costs, we estimate the total processing time for various screening scales on a single core of Xeon Gold 6230R and on a single RTX 2080 Ti. Table S5 summarizes the estimated computational costs for representative screening scenarios. These results demonstrate that on a single core, USP-ddG is practically feasible only for small-to-medium scale screening tasks up to approximately 10^4 mutants. Single-site saturation scans (10^4 mutants) are marginally feasible, while multi-point screening at the 10^5 scale approaches the upper limit of practical application.

Table S5: Estimated computational cost for representative screening scenarios on CPU (single-core) and GPU (single-card) platforms.

Screening Scenario	Mutants	Sites per Mutant	Total Sites	Time per Site (s)	Estimated Time
Small-scale double mutant library	1,000	2	2,000	17.8	9.9 hours
Medium-scale double mutant library	10,000	2	20,000	17.8	4.1 days
Antibody directed evolution	100,000	3	300,000	17.8	61.8 days
Single-site saturation (500 aa)	9,500	1	9,500	23.5	62.0 hours
Double mutant scan (500 aa)	125,000	2	250,000	17.8	51.5 days

Importantly, virtual screening tasks are inherently embarrassingly parallel, as each mutant can be processed independently. Given that FoldX preprocessing on CPU is the primary computational bottleneck, parallelization across multiple CPU cores allows the total wall-clock time to scale inversely with the number of cores. Table S6 illustrates the impact of CPU parallelization on the representative task of screening 100,000 mutants with three mutation sites ($k = 3$) each.

Table S6: Scalability analysis for screening 100,000 mutants ($k = 3$) with parallelization.

CPU Cores	Wall-clock Time	Feasibility Assessment
1	61.8 days	Infeasible
100	14.8 hours	Feasible (single node)
1,000	1.48 hours	Easily feasible (cluster)
10,000	8.9 minutes	Highly efficient

For double mutant scanning is achievable with modest parallelization, completing within half a day on 100 cores. However, full triple mutant scanning pushes the computational limit, requiring large-scale clusters exceeding 100 cores to complete within days. Quadruple mutants and beyond remain computationally prohibitive regardless of parallelization scale, with estimated processing times extending to years even on large clusters, as shown in Table S7.

Further analysis reveals that FoldX preprocessing accounts for over 90% of the total computational cost, constituting the primary bottleneck for large-scale screening. The higher per-site cost observed for single-point mutants compared to multi-point mutants further confirms the presence of fixed initialization overhead that could potentially be amortized through optimized batch processing. Consequently, key directions for improving computational feasibility include developing end-to-end deep learning surrogate models to replace explicit FoldX-based structure preprocessing, reducing per-mutation-site cost to below one second to bring triple and higher-order combinatorial scanning into the feasible range, and optimizing batch processing modes to amortize initialization overhead across multiple mutants.

In summary, USP-ddG achieves a per-mutation-site processing time of approximately 17.8 seconds for multi-point mutations and 23.5 seconds for single-point mutations. With moderate parallelization on 100

Table S7: Computational feasibility for combinatorial mutant scanning (500-residue protein)[a].

Combination Type	Mutants	Sites per Mutant	Total Sites	Single-core Time	100-core Time	Feasibility
Double mutant	1.25×10^5	2	2.5×10^5	51.5 days	12.4 hours	Feasible
Triple mutant	2.07×10^7	3	6.21×10^7	35 years	128 days	Infeasible
Quadruple mutant	2.58×10^9	4	1.03×10^{10}	5,820 years	58.2 years	Infeasible

[a] Here we assume one amino acid substitution per mutated site (e.g., alanine scanning), yielding C(500, n) mutants for n-site combinations.

cores, the method can efficiently support screening tasks at the scale of 10^5mutants, completing within hours to a single day. Full double mutant combinatorial scanning is readily achievable with modest cluster resources, while triple mutant scanning requires substantial parallelization and pushes the boundaries of current computational capacity. The overwhelming dominance of FoldX preprocessing in total runtime highlights the critical need for accelerated surrogate models to enable truly large-scale combinatorial screening across the proteome.

K Error Analysis of Performance Limitations

To better understand the limitations of USP-ddG, we conduct a targeted error analysis to characterize the mutation types and structural contexts where USP-ddG underperforms. We select the protein complex with the lowest prediction performance (PDB ID: 1JTG) as a representative case and use the Mean Absolute Error (MAE) as the evaluation metric.

We first examine the mutation distribution and structural similarity of this complex. Figure S3 presents the TM-score distribution between 1JTG and the training set. The generally low TM-scores indicate limited structural similarity, suggesting that 1JTG lies outside the training distribution. This lack of structurally similar templates likely contributes to the degraded prediction performance. In addition, the distribution of amino acid substitutions in 1JTG is shown in Figure S4, revealing a strong bias toward alanine-scanning mutations.

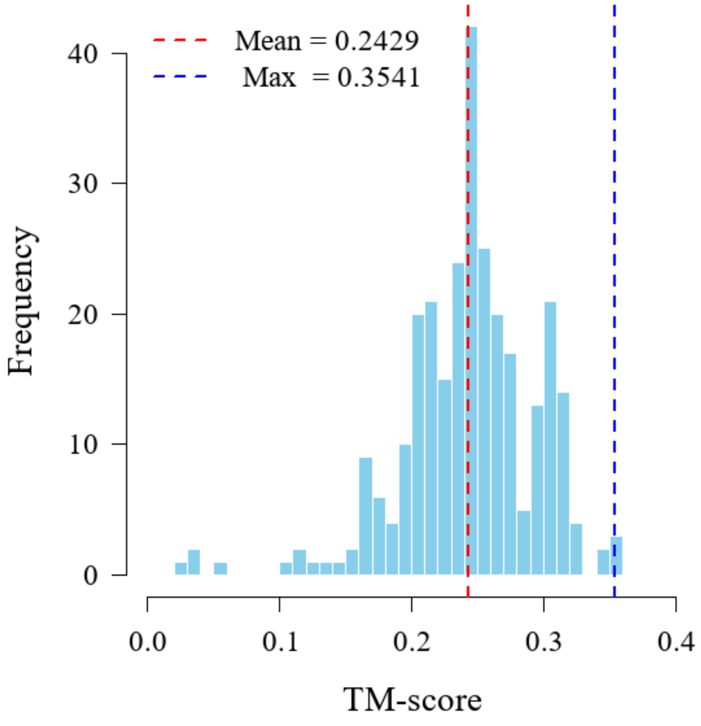

Fig. S3: TM-score distribution between 1JTG and the training set.

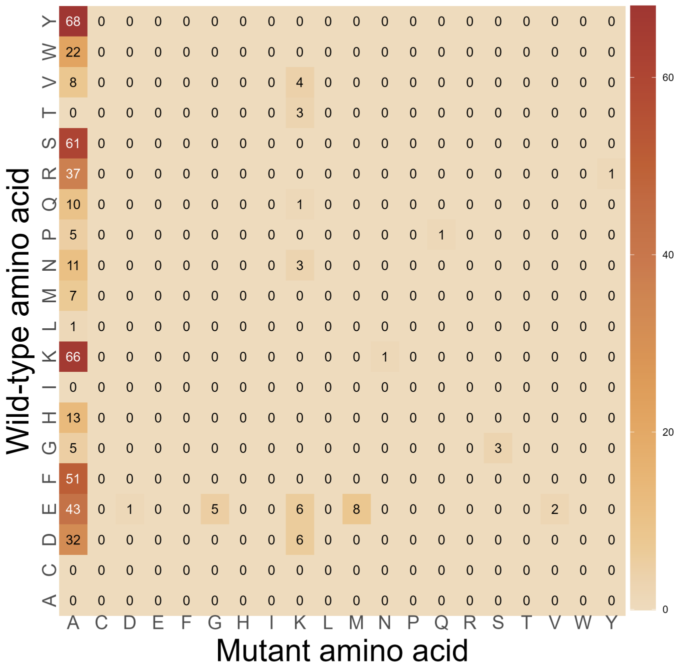

Fig. S4: Distribution of amino acid substitutions in 1JTG with a high prevalence of alanine mutations.

Beyond structural similarity, we also investigate how prediction error scales with mutation complexity. As summarized in Table S8, the error increases substantially with the number of mutation sites. Single-point mutations yield the lowest average error (1.560 kcal/mol), while higher-order mutations exhibit progressively larger errors, reaching 5.837 kcal/mol for quintuple mutations. A similar trend is observed when considering mutation locations. As shown in Table S9, mutations spanning both chains exhibit significantly higher errors than those confined to a single chain, suggesting that simultaneous perturbations to both interacting partners introduce stronger structural and energetic coupling.

Table S8: Prediction error by mutation order.

**Mutation Order**	**Samples**	**MAE (kcal/mol)**	**Std Dev**
Single-point	138	1.560	1.508
Double-point	83	2.660	2.299
Triple-point	37	4.192	2.597
Quadruple-point	15	5.329	3.337
Quintuple-point	2	5.837	6.183

We also compare USP-ddG against a representative energy function method. The table lists the results relative to the energy function baseline method, flex ddG, showing that USP-ddG does not achieve better prediction performance than flex ddG on 1JTG, as illustrated in Figure S5.

K.1 Mutation Types

From the perspective of physicochemical classification (Table S10), mutations involving aromatic residues (Phe and Tyr) exhibit the largest prediction errors, whereas polar uncharged residues show the lowest. Charged residues display intermediate behavior, with Glu and Lys showing relatively higher errors. This

Table S9: Prediction error by mutation chain context.

Mutation Context	Samples	MAE (kcal/mol)	Std Dev
A chain only	56	2.130	1.791
B chain only	109	1.497	1.583
Cross-chain	110	3.640	2.788

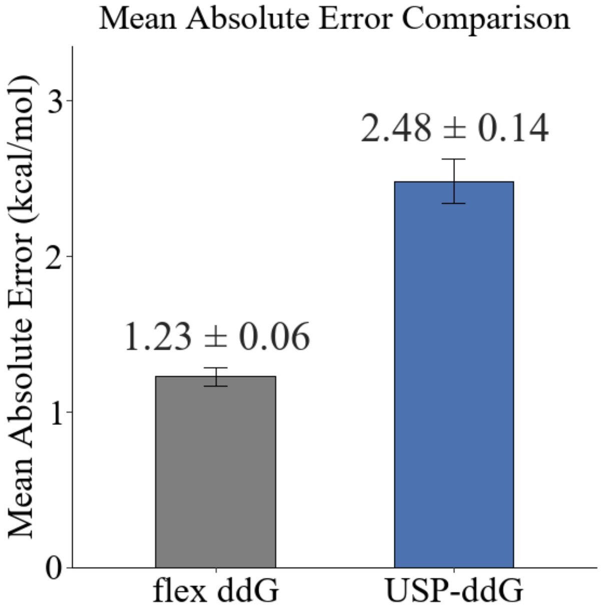

Fig. S5: MAE comparison between USP-ddG and flex ddG predictions for mutations on 1JTG.

pattern suggests that disruptions of aromatic interactions, hydrophobic packing, and electrostatic networks introduce non-linear energetic effects that are difficult to capture. We also present the MAE statistics for each mutation type on the hold out CATH test set, grouped by physicochemical classification. As shown in Table S11, aromatic residues (particularly F and Y) and basic residues (K) exhibit substantially higher prediction errors, with MAE values of 4.145, 3.529, and 2.995 kcal/mol, respectively.

K.2 Structural Contexts

As illustrated in Figure S6, the five mutation sites—EA79, YA80, KB74, FB142, and YB143—are all located at the interface between chains A and B, indicating their potential role in mediating inter-chain interactions. Consistent with this structural distribution, Table S12 shows that mutations at these key interface residues (e.g., positions 79–80 and 142–143) exhibit substantially higher prediction errors, with MAE values exceeding 5 kcal/mol in several cases. These residues likely correspond to energetically critical hotspot positions at the protein–protein binding interface. Mutations at such sites can induce pronounced interface reorganization and non-linear energetic changes, which pose significant challenges for $\Delta\Delta G$ prediction.

K.3 Data Scarcity and Distribution Shift

High-quality experimental $\Delta\Delta G$ data (e.g., SKEMPI v2.0) are limited in size, comprising only 352 PPIs and 7,085 mutations, and cover only a narrow subset of protein space. This limitation restricts the model's ability to learn generalizable features across diverse mutation types and structural contexts. The training data are heavily biased toward specific protein families and mutation types (e.g., alanine scanning), resulting in a

Table S10: Statistical results of mutations grouped by physicochemical classification on 1JTG.

Amino acid	3-letter code	Classification	Count	MAE (kcal/mol)	Std Dev
D	Asp	Acidic (Asp, Glu)	36	1.907	1.330
E	Glu	Acidic (Asp, Glu)	59	4.046	3.142
R	Arg	Basic (Arg, Lys, His)	38	2.506	1.728
K	Lys	Basic (Arg, Lys, His)	63	3.319	2.756
H	His	Basic (Arg, Lys, His)	13	0.731	0.652
F	Phe	Aromatic (Phe, Tyr, Trp)	51	4.995	2.821
Y	Tyr	Aromatic (Phe, Tyr, Trp)	60	4.762	3.129
W	Trp	Aromatic (Phe, Tyr, Trp)	22	0.903	0.580
G	Gly	Nonpolar aliphatic	8	1.027	0.709
L	Leu	Nonpolar aliphatic	1	1.085	-
M	Met	Nonpolar aliphatic	7	1.856	1.474
P	Pro	Nonpolar aliphatic	6	3.691	1.400
V	Val	Nonpolar aliphatic	12	2.110	2.038
N	Asn	Polar uncharged	14	1.229	1.120
Q	Gln	Polar uncharged	11	1.333	1.339
S	Ser	Polar uncharged	49	1.846	1.219
T	Thr	Polar uncharged	3	0.389	0.368

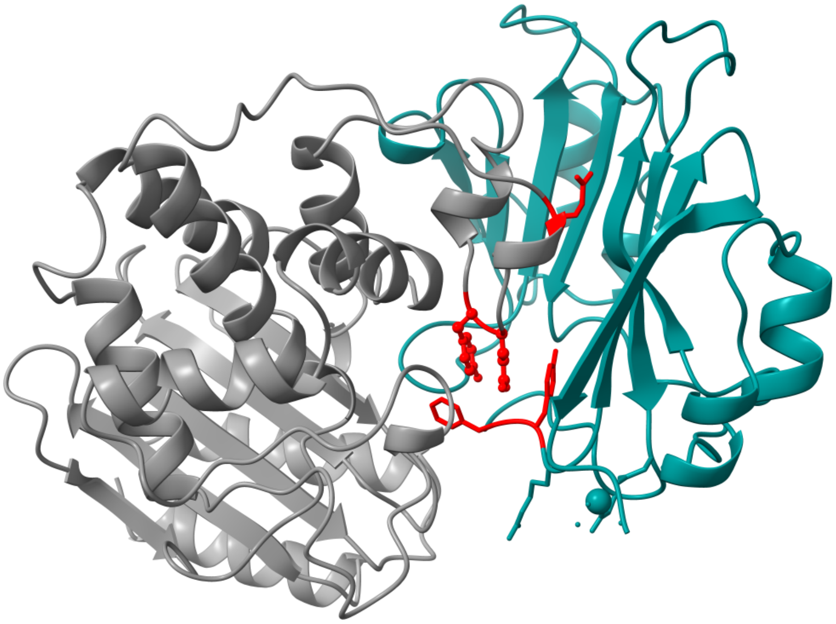

Fig. S6: Visualization of the five mutation sites in 1JTG, and the mutation sites are highlighted in red.

Table S11: Statistical results of mutations grouped by physicochemical classification on hold out CATH test set.

Amino acid	3-letter code	Classification	Count	MAE (kcal/mol)	Std Dev
D	Asp	Acidic (Asp, Glu)	115	2.194	2.028
E	Glu	Acidic (Asp, Glu)	164	2.843	2.602
R	Arg	Basic (Arg, Lys, His)	112	1.873	1.525
K	Lys	Basic (Arg, Lys, His)	114	2.995	2.575
H	His	Basic (Arg, Lys, His)	48	1.169	0.945
F	Phe	Aromatic (Phe, Tyr, Trp)	93	4.145	2.888
Y	Tyr	Aromatic (Phe, Tyr, Trp)	106	3.529	2.976
W	Trp	Aromatic (Phe, Tyr, Trp)	45	1.087	0.808
A	Ala	Nonpolar aliphatic	43	1.903	1.425
G	Gly	Nonpolar aliphatic	27	1.785	1.212
I	Ile	Nonpolar aliphatic	10	1.339	0.937
L	Leu	Nonpolar aliphatic	32	1.875	1.284
M	Met	Nonpolar aliphatic	13	1.808	1.283
P	Pro	Nonpolar aliphatic	14	2.125	1.772
V	Val	Nonpolar aliphatic	52	1.818	1.356
N	Asn	Polar uncharged	62	1.729	1.452
Q	Gln	Polar uncharged	26	1.425	1.126
S	Ser	Polar uncharged	100	1.478	1.176
T	Thr	Polar uncharged	51	1.425	1.247
C	Cys	Polar uncharged	4	1.907	1.724

Table S12: Statistical results for key mutation sites.

Mutation site	Count	MAE (kcal/mol)	Std Dev
YA80	17	8.183	1.708
EA79	30	6.132	2.792
KB74	40	3.940	3.211
FB142	45	5.601	2.416
YB143	31	4.764	2.823

distribution shift when generalizing to unseen mutations or proteins with different evolutionary backgrounds. This shift inherently caps the achievable correlation coefficients on hold-out test sets. Incorporating larger and more diverse experimental datasets, along with improved generalization techniques, represents a key direction for future work to further enhance the accuracy of $\Delta\Delta G$ prediction.

Overall, these results indicate that USP-ddG tends to underperform in scenarios involving low structural similarity, multi-point mutations, cross-chain mutations, aromatic residue substitutions, and mutations at interface hotspot residues. Such challenging cases are difficult to capture under the current local perturbation and modeling assumptions.

References

1. Atkins, P.W., de Paula, J., Keeler, J.: Atkins' Physical Chemistry. Oxford University Press, New York, NY, United States of America (2023)

2. Bachlechner, T., Majumder, B.P., Mao, H.H., Cottrell, G., McAuley, J.J.: ReZero is all you need: fast convergence at large depth. In: Proceedings of the Thirty-Seventh Conference on Uncertainty in Artificial Intelligence, UAI 2021, Virtual Event. Proceedings of Machine Learning Research, vol. 161, pp. 1352–1361. AUAI Press (2021), https://proceedings.mlr.press/v161/bachlechner21a.html
3. Dauparas, J., Anishchenko, I., Bennett, N., Bai, H., Ragotte, R.J., Milles, L.F., Wicky, B.I., Courbet, A., de Haas, R.J., Bethel, N., et al.: Robust deep learning based protein sequence design using ProteinMPNN. Science **378**(6615), 49–56 (2022)
4. Feng, S., Ni, Y., Lan, Y., Ma, Z.M., Ma, W.Y.: Fractional denoising for 3d molecular pre-training. In: Proceedings of the Fortieth International Conference on Machine Learning. pp. 9938–9961. PMLR, Honolulu, Hawaii, USA (2023)
5. Glorot, X., Bordes, A., Bengio, Y.: Deep sparse rectifier neural networks. In: Proceedings of the Fourteenth International Conference on Artificial Intelligence and Statistics. pp. 315–323. AISTATS 2011, Fort Lauderdale, FL, USA (2011)
6. Ingraham, J., Garg, V., Barzilay, R., Jaakkola, T.: Generative models for graph-based protein design. Advances in neural information processing systems **32** (2019)
7. Jiao, X., Mao, W., Jin, W., Yang, P., Chen, H., Shen, C.: Boltzmann-aligned inverse folding model as a predictor of mutational effects on protein-protein interactions. In: Proceedings of the Thirteenth International Conference on Learning Representations. pp. 56810–56827. ICLR 2025, Singapore (2025). https://doi.org/10.48550/arXiv.2410.09543
8. Jin, W., Chen, X., Vetticaden, A., Sarzikova, S., Raychowdhury, R., Uhler, C., Hacohen, N.: DSMBind: SE(3) denoising score matching for unsupervised binding energy prediction and nanobody design. In: Proceedings of the NeurIPS 2023 Generative AI and Biology (GenBio) Workshop. NeurIPS 2023, New Orleans, LA, USA (2023). https://doi.org/10.1101/2023.12.10.570461
9. Liu, S., Zhu, T., Ren, M., Yu, C., Bu, D., Zhang, H.: Predicting mutational effects on protein-protein binding via a side-chain diffusion probabilistic model. In: Advances in Neural Information Processing Systems 36. vol. 36, pp. 48994–49005. NeurIPS 2023, New Orleans, LA, USA (2023)
10. McPartlon, M., Xu, J.: An end-to-end deep learning method for protein side-chain packing and inverse folding. Proceedings of the National Academy of Sciences **120**(23), e2216438120 (2023)
11. Starr, T.N., Greaney, A.J., Hannon, W.W., Loes, A.N., Hauser, K., Dillen, J.R., Ferri, E., Farrell, A.G., Dadonaite, B., McCallum, M., Matreyek, K.A., Corti, D., Veesler, D., Snell, G., Bloom, J.D.: Shifting mutational constraints in the SARS-CoV-2 receptor-binding domain during viral evolution. Science **377**(6604), 420–424 (2022). https://doi.org/10.1126/science.abo7896
12. Van Kempen, M., Kim, S.S., Tumescheit, C., Mirdita, M., Lee, J., Gilchrist, C.L., Söding, J., Steinegger, M.: Fast and accurate protein structure search with foldseek. Nature biotechnology **42**(2), 243–246 (2024)
13. Xu, J., Sun, X., Zhang, Z., Zhao, G., Lin, J.: Understanding and improving layer normalization. In: Advances in Neural Information Processing Systems 32: Annual Conference on Neural Information Processing Systems 2019, NeurIPS 2019, December 8-14, 2019, Vancouver, BC, Canada. pp. 4383–4393 (2019), https://proceedings.neurips.cc/paper/2019/hash/2f4fe03d77724a7217006e5d16728874-Abstract.html
14. Yu, G., Bi, X., Ma, T., Li, Y., Wang, J.: CATH-ddG: Towards robust mutation effect prediction on protein–protein interactions out of CATH homologous superfamily. Bioinformatics **41**(Supplement_1), i362–i372 (2025). https://doi.org/10.1093/bioinformatics/btaf228
